# IST Nurse^®^: development and validity evidence of a mobile application as a support for the clinical management of Sexually Transmitted Infections*

**DOI:** 10.1590/1518-8345.7207.4317

**Published:** 2024-09-02

**Authors:** Leilane Barbosa de Sousa, Ismael Moreira de Sousa, Edmara Chaves Costa, Ana Paula Fragoso de Freitas, Lydia Vieira Freitas dos Santos, Adriana Gomes Nogueira Ferreira

**Affiliations:** 1 Universidade da Integração Internacional da Lusofonia Afro-Brasileira, Redenção, CE, Brazil.; 3 Faculdade Rodolfo Teófilo, Curso de Graduação em Enfermagem, Fortaleza, CE, Brazil.; 4 Universidade Federal do Maranhão, Imperatriz, MA, Brazil.

**Keywords:** Mobile Applications, Information Technology, Disease Management, Sexually Transmitted Diseases, Nursing Care, Public Health

## Abstract

**Objective:**

to develop and evaluate the validity evidence of a mobile application to support nurses in the clinical management of sexually transmitted infections.

**Method:**

methodological study conducted in four steps: analysis and definition of requirements; content definition; computational representation with system design; and coding with testing and refinement. In the first steps, nurses with expertise in the subject participated, and in the last, professionals with education in information and communication technology. Data analysis was performed by calculating the Content Validity Index (CVI), considering the minimum agreement value of 0.78. To confirm the viability of the CVI, the binomial test was used through the R software. Variables with p > 0.05 indicated agreement between the judges.

**Results:**

the CVI was 0.98 for content, 1.0 for usability and 0.85 for functional performance, showing that the developed application has high validity.

**Conclusion:**

it is believed that the IST Nurse^®^ application represents an important technological tool in strengthening evidence-based nursing care. Intervention studies are therefore suggested.

## Highlights

**(1)** Lack of applications related to STI capable of supporting clinical management.

**(2)** Strengthening PHC in the early identification and treatment of STI.

**(3)** Useful product for clinical practice, education and standardization of nurses’ actions.

**(4)** Reduction of errors in professional conduct, increasing the quality of care.

**(5)** Mobile technologies democratize access to information, with good cost-effectiveness.

## Introduction

The clinical management of sexually transmitted infections (STI) is still a challenge for health professionals, including nurses. Ineffective clinical evaluation compromises the breaking of the transmission chain of these infections, increasing the number of cases. In the meantime, clinical reasoning is an essential element for the decision-making process and favors early diagnosis and treatment, these being essential factors for the control and surveillance of STI^1^ ^)-(^ ^2^.

Detecting and treating STI early reduces the risk of spread, which highlights the need to find methods to improve care for people with STI, especially in the context of HIV/AIDS^3^. Therefore, nurses can benefit from technological tools that can support nursing care in gynecology, urology and andrology, covering the different populations susceptible to STI.

The use of eHealth technologies and their mHealth component facilitates access and dissemination of information, allowing interaction and sharing of knowledge between people in different geographic locations^4^. The ability to generate, store and share pertinent information at a fast pace makes technology devices attractive tools that are increasingly used in the routine of anyone who uses these resources^5^.

However, there is a gap in the production and availability of applications that address the clinical management of STI. In a narrative review of the literature in January 2024 for mobile applications that address the STI theme, 38 applications were found for Android and three for the iPhone Operating System (iOS); however, the identified technologies are not aimed at the clinical management of STI.

It is believed that a mobile application developed based on comprehensive care protocols and algorithms that guide nurses towards clinical reasoning may present validity evidence to support assistance to people with signs and/or symptoms of STI. Thus, the present study aimed to develop and evaluate the validity evidence of a mobile application to support nurses in the clinical management of STI.

### Method

### Type of study

This is a methodological study based on the development and analysis of validity evidence of an application for mobile devices on the clinical management of STI by nurses. In summary, this type of study makes it possible to investigate the methods of obtaining, systematizing and analyzing data for the construction of an instrument^6^.

The study was developed in two phases. Phase 1 was the development of the application in four steps: (1) analysis and definition of requirements, (2) definition of content, (3) computational representation with prototype projection, and (4) coding with testing and refinement. In phase 2, the search for validity evidence of the application occurred, in terms of content, functional performance and usability.

### Location and period of study

The study was carried out in the city of Redenção, Ceará (CE), Brazil, between September 2021 and June 2022.

### Population

The study included nurses working in the Family Health Strategy (FHS) (definition of requirements for the application and analysis of usability evidence), nurses working in teaching at higher education level (analysis of content validity evidence) and professionals with education in information and communication technology (analysis of functional performance validity evidence).

### Sample definition

The invitation to participate in the study was initially made via e-mail, located through research on the Lattes Platform of the *Conselho Nacional de Desenvolvimento Científico e Tecnológico* (CNPq). The body of the e-mail offered a summary containing the title of the research, objectives and aspects of the method, as well as the Free and Informed Consent Form for providing acquiescence. This strategy, however, was unsuccessful.

Therefore, all participants were selected by non-probability sampling, using the reference network technique^7^. This technique occurred through the selection of key informants called seeds, who located some people with the necessary profile for the research. These nominated people were asked to nominate new people with the desired profile. Communication began via WhatsApp, from where a video was sent with the same information contained in the email.

#### Phase 1: Application development

To define nurses, the User-Centered Design method^8^ was considered, in which mutual participation/collaboration between application users is established, with the use of the focus group technique being considered effective at this step. The number of participants was defined as between six and twelve per group, according to the reference^9^.

Via WhatsApp, potential participants were contacted and guided through voice messages about the study proposal, including ethical issues for participating in the research.

After 23 days of starting contact with potential research participants, indicated by key informants, it was possible to select 15 participants. Given the incompatibility of available times to participate in the same group session, it was necessary to divide the professionals into two groups, one consisting of nine nurses and the other of six.

#### Phase 2: Validity evidence of the application 

To select judges to analyze functional performance validity evidence, some defining characteristics were considered based on the proposal^10^, which were adapted for this study. And for the analysis of usability evidence, contact was resumed with the participants of the two focus groups.

Regarding the ideal number of judges, there is no consensus in the literature. Thus, it was defined based on recommendations^7^, which propose a minimum number of six judges. In addition to this recommendation, it is advisable to use an odd number of judges to avoid a tie in opinions^7^. This recommendation was adopted for the analysis of content and usability evidence. Due to the difficulty in finding judges available to analyze functional performance validity evidence, and based on a specific reference for software evaluation, it was found that this analysis works well with a quantity of three to five experts^11^.

Seven judges were selected for content validity evidence, three for functional performance, and seven for the analysis of usability evidence.

### Selection criteria

#### Phase 1: Application development

Nurses working in the FHS with at least one year of experience were included, since, from their daily work with the person/family/group, they have experience in assisting people with STI. The temporal determination is related to the authors’ perception of a minimum period for the experience in the practice of clinical management to allow sufficient property to point out the potentialities and weaknesses of their performance in this field. Those who were on vacation or away on some type of leave were excluded.

#### Phase 2: Validity evidence of the application

Nurse researchers/professors with experience in the area of interest were considered judges for the analysis of content evidence: sexual health, STI and/or health technologies. Those who did not reach at least five points by applying the adapted criteria^10^ were excluded, namely: being a PhD or a master with expertise in the area of interest.

For the analysis of usability validity evidence, nurses who collaborated in the requirements definition step, in phase 1 of the study, participated as judges. The inclusion and exclusion criteria have already been explained and justified previously.

To analyze the functional performance validity evidence, professionals with education and experience in the areas of software engineering, computer science and systems analysis and development were included, and those who did not meet the adapted criteria^10^ were excluded, namely: having a diploma degree in Information and Communication Technology; have professional experience in information/communication/computing technology for a minimum period of two years; and have experience in developing and implementing systems.

### Study phases

#### Phase 1: Application development

Four steps were followed using incremental and interactive method: (1) analysis and definition of requirements; (2) definition of content; (3) computational representation with system design; and (4) coding with testing and refinement^11^ ^)-(^ ^14^.

In the analysis and definition of requirements, we sought to mainly understand the knowledge and actions of nurses in relation to the clinical management of STI. This step allowed access to the nurses’ demands regarding what the application would need to offer.

After identifying the nurses’ demands, a partnership was sought with a team with experience in software analysis and development, led by a research professor with a PhD in Informatics, from the *Universidade Federal do Maranhão*. The team and one of the authors of the proposal held six meetings to define the desired functionalities for the application and gather requirements to later use the prototyping technique^15^. The same author was qualified by the partner team to develop the first version of the application prototype using the Figma platform^16^, in which basic and functional aspects of the system were defined, such as interaction flows, button location, text and image mapping.

The content definition step aimed to gather the best scientific evidence on the proposed topic, and it was decided to use two protocols from the *Ministério da Saúde* as a base reference^1^ ^),(^ ^17^. These are the most current Brazilian references on the subject.

The computational representation with system design step consisted of formatting the content in a suitable format for subsequent coding in Java. This resulted in version 1 of the prototype, which incorporated the textual body formulated from the analysis and definition of requirements, following logical operating flows. The application logo was created using Canva software^18^.

After evaluation of version 1 by the development team, version 2 was structured based on projects made available by the Figma community^16^, following design and Human-Computer Interface (HCI) standards.

In the coding with testing and refinement step, the application was developed in modules, allowing quality testing as each module was implemented. This facilitated early fault detection for quick fixes.

For coding, the Javascript programming language was used with the React Native framework, allowing the creation of applications for Android and iOS. Version control was done with the Git and Github tools, and package installation was performed with npm and yarn.

After coding, the application was installed on an Expo server in the cloud and a link and a Quick Response Code were generated for access. This allowed researchers and judges involved in the study to access the application for testing and refinement.

#### Phase 2: Validity evidence of the application

It occurred in three fields: content, functional performance and usability. To define content judges, a set of proposed requirements was adapted^10^.

### Instruments used and data collection

#### Phase 1: Application development

The focus group sessions were carried out remotely and synchronously, and were recorded via institutional email from one of the authors for later retrieval of the statements. Triggering questions were used during the meetings by an author who moderated the moment, which made group discussion possible.

The recorded material was revisited for consideration, which made it possible to define the professionals’ needs and define the content that should compose the application.

Once the demands of nurses in the clinical management of STI were identified, the relevant content was sought in the manual and protocol made available by the *Ministério da Saúde* on the subject^1^ ^),(^ ^17^.

#### Phase 2: Validity evidence of the application

To content validity evidence, an adapted instrument was used^19^, which includes items distributed in the following blocks: objectives and content (purposes and goals or ends that one wishes to achieve through practice with technology) and structure and functionality (way of presenting the guidelines and practicality in the functions).

In the process of analyzing functional performance validity evidence, an instrument was used based on the Product Quality Model of the International Organization for Standardization/International Electrotechnical Commission (ISO/IEC) 25010 standard, which specifies eight quality characteristics^20^.

To analyze usability validity evidence, the Smartphone Usability Questionnaire (SURE) scale was used, with the aim of enabling the end user to evaluate the application in a subjective way directly observed during the test^21^.

For each item of the instruments and criteria evaluated, a score of 1 - inadequate, 2 - partially adequate, 3 - adequate, 4 - completely adequate and 5 - not applicable, was assigned.

The judges participating in the three modalities of the validity evidence analysis process received the instruments in form via email and via WhatsApp message. Spaces were provided in the instruments for judges to make comments and suggestions about each item evaluated. After 14 days, everyone’s answers were available.

### Data processing and analysis

A synchronous remote meeting was held with each focus group. The details of the study were presented and the triggering questions were asked. The speeches recorded by voice recording and texts were transcribed and processed. The speeches generated by the meetings with the focus groups were analyzed following the collective subject discourse method^22^. As a tool to assist in this analysis, the IRaMuTeq software was used.

In the analysis process, the Content Validity Index (CVI) was used to measure the percentage of agreement for each item individually. The CVI was calculated by the sum of items 3 (adequate) and 4 (inadequate) divided by the total number of responses, considering a minimum agreement value of 0.78^23^. In case of lower agreement, the item would be modified according to suggestions made by the judges.

To confirm the viability of the CVI, the binomial test was also used by processing the binom.test command (n° of successes, total n°, p=05) in the R software: here it was tested whether the success proportion observed in the sample belonged to a population with a certain *p* value, in which *p* values greater than 0.05 indicated agreement between the judges, not being statistically lower than 85%^24^.

### Ethical aspects

The research was approved by the Ethics and Research Committee of the *Universidade da Integração Internacional da Lusofonia Afro-Brasileira* under opinion number 4.889.214.

## Results

A word cloud and a similarity tree were generated by IRaMuTeq and, based on their assessment, thematic categories were formed following the collective subject discourse analysis method. These categories were important for the content definition process.

It was verified through focus groups that the most relevant classes for the content of the application are: difficulties related to the professional’s knowledge about management, recognition of symptoms and lack of adherence of sexual partners to treatment, use of protocols of the *Ministério da Saúde* as a consultation for clinical management, and the practicality and ease offered by technologies such as mobile applications to guide appropriate treatment for STI.

These classes were the basis for the schematization of the theoretical content of the application, with the main references being the *Protocolo Clínico e Diretrizes Terapêuticas para Atenção Integral às Pessoas com IST* ^1^ and the *Caderno de Atenção Básica 18: HIV/Aids, Hepatites Virais, Sífilis e outras IST* ^17^, both from the *Ministério da Saúde*.

Following the React Native standard, 121 screens were created, organized into subcategories of folders according to STI and sections of the application. For example, within the “Queries” folder there are subfolders corresponding to each infection. This structure was defined to facilitate the organization of the files that compose the system, in addition to allowing their subsequent maintenance.

The application does not use any back-end for data processing, it is only an information display and navigation application. Therefore, all screens developed are static, presenting information related to STI and the possibility of navigation (going back or forward on the screens). In addition to the screens referring to the clinical management of STI, others were developed about the project creators, developers and references.

After the entire development process, in total approximately 10,000 lines of code were written, using 54 images. Then, the customer with the Expo application - for testing and refinement - can, through the link or Quick Response Code, open the cloud system on their cell phone. This was done due to the need to run the system on a cell phone with Android and iOS operating systems, and this operating system does not have support and installation packages.

Just as it was designed during prototyping, the application follows a flow that was codified at this step. This flow follows commands defined based on the content used, with computational intelligence applied to the STI clinical management process.

The coded version of the system, created using the Javascript programming language used by the *React Native* framework, presents an interactive application, following the flow described previously, being easy to use and navigate, containing textual information and images, with a scientific basis, dark screen background with white texts and green commands.

**Figure 1:**
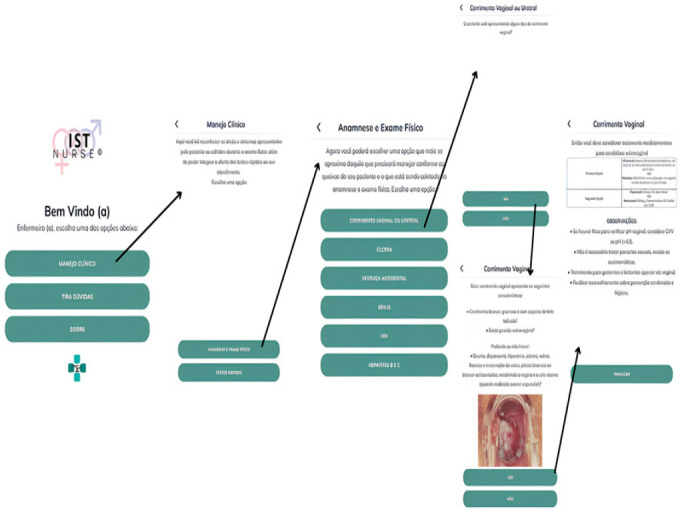
Flow of application steps from home screen to directing to the recommended treatment for vaginal discharge. Redenção, CE, Brazil, 2022

The application’s initial screens are presented in Figure 1, which shows the path from the Home screen to the therapeutic approach based on clinical management. 

### Analysis of content validity evidence

The seven nurse judges, with experience in teaching at a higher education level, as well as everyone else, participated in a research group. The areas of activity were sexual and reproductive health (n=2), family health (n=3) and collective health (n=2), and the average time working in the areas was 8.43 years. All obtained a score higher than five, concerning the sum of the scores attributed by the set of requirements.

Four (57.2%) judges had a master’s degree, and three (42.9%) had a doctorate. One of the judges supervised a dissertation, four supervised a monograph or course completion work. They all had publications involving topics of interest. Three judges combined teaching and research, and three have published research on the topics of STI and Health Technology.

According to Table 1, the judges’ assessment showed an average of 4 (completely adequate) for most of the items evaluated, with an average of 3.71 for the item that evaluates the organization of information. Therefore, the items presented CVI with statistically significant values, with a proportion of agreement between judges greater than 85% (*p*>0.05).

**Table 1:** Distribution of judges’ responses on the content presented by the IST* Nurse^®^ application. Redenção, CE, Brazil, 2022

N°	Questions	min^†^	m^‡^	max^§^	std dev^||^	CVI^¶^	p**
1	Does the application maintain coherence with the needs of nurses regarding clinical reasoning for the management of STI*?	4	4	4	0	1	1
2	Does the application promote systematization for the clinical management of STI*?	4	4	4	0	1	1
3	Can the application circulate in the scientific community in the area of clinical management of STI*?	4	4	4	0	1	1
4	Is the application material suitable for supporting nurses during the clinical management of STI*?	4	4	4	0	1	1
5	Is the information organized in a clear and objective way?	3	3.71	4	0.49	1	1
6	Is the information presented consistent with scientific evidence?	4	4	4	0	1	1
7	Is there a logical sequence of the proposed content?	4	4	4	0	1	1
8	Is the material appropriate in terms of the sociocultural level of the proposed target audience?	4	4	4	0	1	1
9	Is the information well-structured in terms of agreement and spelling?	2	3.29	4	0.95	0,76	0,71
10	Does the writing style match the level of knowledge of the proposed target audience?	3	3.86	4	0.38	1	1
11	Are the illustrations expressive and sufficient?	3	3.43	4	0.53	1	1
12	Is the number of steps adequate?	3	3.86	4	0.38	1	1
13	Are the size of items and topics appropriate?	3	3.86	4	0.38	1	1
14	Do the themes portray the key aspects that must be paid attention to?	3	3.86	4	0.38	1	1
15	Does the application provide nurses with sufficient requirements aimed at people with STI^*^ for the clinical management of STI*?	3	3.86	4	0.38	1	1
16	Is the application suitable for use by any nurse who works with care for people with STI*?	3	3.86	4	0.38	1	1

*STI = Sexually transmitted infection; ^†^min = Minimum score; ^‡^m = Mean; ^§^max = Maximum score; ^||^std dev = Standard deviation; ^¶^CVI = Content Validity Index; **p = Binomial test

The only item that presented a low proportion was nine, referring to content agreement and spelling, which received an average of 3.29. The considerations highlighted by the judges were met through spelling review and correction.

Errors in the application were attributed to typing mistakes and the platform not recognizing certain terms, resulting in the automatic generation of similar words. The judges praised the synthesis and completeness of the content, the systematization and the agility of the application, but suggested more illustrations to improve clinical management. They also highlighted the application’s efficiency in addressing key aspects of the content, with suggestions to include information about hepatitis and the immunological window.

Application adjustment was made based on this feedback. With a standard deviation indicating homogeneity in the sample and a Content Validity Index (CVI) of 0.98, the application shows validity evidence and is considered useful for nurses dealing with STI.

### Analysis of usability validity evidence

Seven judges participated in this step, all nurses graduated between 2015 and 2020, with an average experience of 3.29 years in the FHS. The majority (85.71%) were female, while only one was male (14.29%). Six were specialists (85.71%) and one had a master’s degree (14.29%).

The application evaluation followed the SURE scale^21^ for analyzing usability validity evidence. The judges’ responses indicated that some questions were considered positive in practice, but they disagreed with the statements proposed by the scale, and were not used to calculate the CVI.

According to Table 2, the average evaluation of the questions on the previously mentioned scale varied between 3.57 and 3.86, demonstrating homogeneity in the sample regarding the suitability of the application for the clinical management of STI. The CVI was 1.0, reflecting unanimous positive evaluation of the application by nurses. The judges’ statements corroborated the data, showing satisfaction with the appearance, usability and theoretical content of the application, highlighting its practicality and ability to facilitate nurses’ practice with people with STI. 

**Table 2:** Distribution of judges’ responses on usability of the IST* Nurse^®^ application. Redenção, CE, Brazil, 2022

N°	Questions	min^†^	m^‡^	max^§^	std dev^||^	CVI^¶^	p**
1	I found it easy to insert data into this application. For example, using QR code, list of options, etc.	3	3.57	4	0.53	1	1
2	When I make a mistake, it’s easy to correct it.	3	3.57	4	0.53	1	1
3	I found the help/tips given by the application to be useful.	3	3.71	4	0.49	1	1
4	It was easy to find the information I needed.	3	3.71	4	0.49	1	1
5	I felt in command using this application.	3	3.71	4	0.49	1	1
6	I thought the time it took to complete the tasks was adequate.	3	3.71	4	0.49	1	1
7	It was easy to learn how to use this application. (To answer this question, you should only consider using the IST* Nurse^®^ application, not the installation process).	3	3.71	4	0.49	1	1
8	The sequence of actions in the application corresponds to the way I normally perform them, for example, the order of buttons, data fields, etc.	3	3.57	4	0.53	1	1
9	It is easy to do what I want using this application.	3	3.71	4	0.49	1	1
10	It was easy to navigate the application’s menus and screens.	3	3.86	4	0.38	1	1
11	The application meets my needs.	3	3.86	4	0.38	1	1
12	I would recommend this applicationto others.	3	3.86	4	0.38	1	1
13	Even in a hurry, I would be able to perform tasks in this application.	3	3.86	4	0.38	1	1
14	I found the application to be consistent. For example, all functions can be performed in a similar way.	3	3.71	4	0.49	1	1
15	It is easy to remember how to do things on this application.	3	3.86	4	0.38	1	1
16	I would use this application frequently.	3	3.71	4	0.49	1	1
17	The organization of menus and action commands (such as buttons and links) is logical, allowing to easily find them on the screen.	3	3.86	4	0.38	1	1
18	I was able to complete the tasks successfully using this application.	3	3.86	4	0.38	1	1
19	I enjoyed using this application.	3	3.86	4	0.38	1	1
20	The application offers all the information necessary to complete tasks in a clear and understandable way.	3	3.86	4	0.38	1	1
21	I found the application very complicated to use.	1	1	1	0	0	0
22	The symbols and items are clear and intuitive.	3	3.71	4	0.49	1	1
23	I found the texts easy to read.	3	3.86	4	0.38	1	1
24	I found the application unnecessarily complex. I had to remember, research or think a lot to complete the tasks.	1	1	1	0	0	0
25	The terminology used in texts, labels, titles, etc., is easy to understand.	3	3.71	4	0.49	1	1
26	I would need the support of a person to use this application.	1	1.29	2	0.49	0	0
27	I felt comfortable using this application.	3	3.71	4	0.49	1	1
28	The application behaved as I expected.	3	3.71	4	0.49	1	1
29	I found using this application frustrating.	1	1	1	0	0	0
30	I found the application’s various functions to be well integrated.	3	3.57	4	0.53	1	1
31	I felt very confident using this application.	3	3.86	4	0.38	1	1

*STI = Sexually transmitted infection; ^†^min = Minimum score; ^‡^m = Mean; ^§^max = Maximum score; ^||^std dev = Standard deviation; ^¶^CVI = Content Validity Index; **p = Binomial test

### Analysis of functional performance validity evidence

Three judges participated in this step, with education in Computer Science (n=2) and Information Systems (n=1), the latter being female. The education year is between 2015 and 2021, with an average of 3.67 years of experience in the education area.

Table 3 presents the results of the evaluation of the judges who carried out the analysis of the functional performance validity evidence. The question evaluation averages were between 3 and 4, which indicates that the judges agreed that the application’s functionalities are adequate or completely adequate. The standard deviation corroborated this, ranging from 0 to 0.58. The CVI was statistically significant, and, in addition, the *p* value for all items shows a 100% agreement proportion between the judges.

**Table 3:** Distribution of judges’ responses on functional performance of the IST* Nurse^®^ application. Redenção, CE, Brazil, 2022

N°	Questions	min^†^	m^‡^	max^§^	std dev^||^	CVI^¶^	p**
1	Are the available functions sufficient to carry out the tasks proposed by the application?	4	4	4	0	1	1
2	Is the language used in the application understandable?	3	3.67	4	0.58	1	1
3	Is the application consistent with the target audience it is intended for?	4	4	4	0	1	1
4	Is the amount of information placed on each screen appropriate for the target audience?	3	3.67	4	0.58	1	1
5	Are the colors used with balance, that is, are they well distributed, thus avoiding visual pollution?	3	3.67	4	0.58	1	1
6	By using the application, is it possible to obtain information to achieve your primary objective?	3	3.67	4	0.58	1	1
7	Does it invite or instigate the user to systematic reasoning?	4	4	4	0	1	1
8	Is the information contained in the application coherent?	4	4	4	0	1	1
9	Is it attractive to the user?	4	4	4	0	1	1
10	Is it easy to understand the concepts used?	4	4	4	0	1	1
11	Are the functions easy to learn to use?	4	4	4	0	1	1
12	Are the presentations of interface functions (icons, menus...) easy to understand?	3	3.67	4	0.58	1	1
13	Is it easy to operate and control the operation?	4	4	4	0	1	1
14	Is the application consistent with what is expected in relation to what is scientifically proposed?	4	4	4	0	1	1
15	In the presence of errors, does the application allow recovery of the data already provided?	3	3	3	NA^††^	1	1
16	Does it prevent unauthorized, accidental or deliberate access to data programs?	3	3.5	4	0.71	1	1
17	Does it promote user integrity?	4	4	4	0	1	1
18	Is it easy to find faults when they occur?	3	3	3	NA^††^	1	1
19	Is it easy to adapt to other environments without applying other actions or means than those provided for this purpose in the software considered?	3	3.67	4	0.58	1	1
20	Is the application easy to install?	3	3.67	4	0.58	1	1
21	Is it good enough to be replaced?	4	4	4	NA^††^	1	1
22	Does it issue some form of feedback when the user follows a negative line of reasoning?	4	4	4	0	1	1
23	Does it offer a summary of user performance at the end of use?	4	4	4	0	1	1

*STI = Sexually transmitted infection; ^†^min = Minimum score; ^‡^m = Mean; ^§^max = Maximum score; ^||^std dev = Standard deviation; ^¶^CVI = Content Validity Index; **p = Binomial test; ^††^NA = Not applicable

Regarding questions 15 and 17, considering that one of the judges pointed out the need for patient registration in the application, this is something that, by decision of the researchers, was disregarded due to the protection of patient data, since the application will be in use on the nurse’s cell phone, and not on a device exclusive to the service. The application aims to guide the clinical management of STI with regard to the propaedeutic characteristics evidenced during the nursing consultation, which will provide support for critical and reflective thinking about the clinical assessment. However, other personal data must be considered by the professional for recording in the service’s own documents.

About questions 16 and 23, the considerations were taken to the team that developed the system to increase a form of restricted access for professionals who have the IST Nurse^®^ application on their smartphone, as well as offering a summary of the steps followed by the user and the conduct adopted.

From questions 15 to 18, and 21 to 23, some judges marked the option “NA” as not applicable. This conveys the information that the evaluator would not like to answer that question as they consider it inappropriate for the objective of the evaluation process. In view of this, in the questions in which this option was selected by a judge, they were not considered as a respondent for the calculation of the CVI.

The CVI presented a value of 0.85, representing an excellent level of agreement among experts, and reflects that the application has adequate or completely adequate commands and functions to accomplish what it proposes.

At the end of the presentation of these data, Figure 2 is presented, which shows the judges’ suggestions for improving the application, as well as indicating whether or not the suggestions were accepted by the authors, and in what way.

**Figure 2:** Suggestions from the judges and the authors’ decision on changes to the first template of the application. Redenção, CE, Brazil, 2022

Suggestion	Decision
**Analysis of content evidence**	
Better illustrate the home screen.	Accepted. The logo was modified to better relate to the purpose of the application.
Insert more images to help the nurse better identify/suspect during management.	Accepted. New images were inserted for the presented clinic of infections.
Improve image quality.	
Improve information on the conduct of syphilis management, especially regarding serological scar.	Accepted. Content was added to guide nurses regarding the need to request tests and conduct to monitor syphilis cases.
Address the immunological window in the issue of rapid tests.	Accepted. Information was inserted to request a new collection within 30 days in case of suspicion or negative results.
Improve quick test images.	Accepted. New images were inserted to assist in the interpretation of rapid test results.
Insert treatment for anogenital warts.	Accepted. The treatment indication was inserted.
**Analysis of functional performance evidence**	
Facilitate the installation process by entering one of the stores.	Not accepted. The application will only be made available in stores after application in an effectiveness evaluation study.
**Analysis of usability evidence**	
Insert a link in the application that leads to the notification.	Not accepted. This possibility had been considered during prototyping, but due to guidance from the software development team, it was not included.

## Discussion

The development of a technology aimed at the daily lives of nurses needs to consider the demands they present. During the development of the IST Nurse^®^ application, the online focus group strategy proved to be effective in identifying themes that should make up the content of the technological tool, as it allowed dialogue with professionals working in different cities and the verification of needs and priorities within the scope of clinical management of STI. A similar study, which also used the online focus group as a situational diagnosis strategy, highlighted that this strategy contributed significantly to the construction of knowledge in the health area^25^.

The demands listed from the dialogue with the nurses constituted the starting point for producing the application’s content. Along this path, the *Protocolo Clínico e Diretrizes Terapêuticas para Atenção Integral às Pessoas com Infecções Sexualmente Transmissíveis* (PCDT-IST)^1^ was configured as a relevant reference for the basis of data flow in the application, as it presents information based on scientific evidence about the characteristics of STI, and criteria for diagnoses and management, including not only pharmacological treatment, but also guidelines aimed at prevention and control, which are essential to nursing care. Using the PCDT-IST, it was possible to develop content aimed at conducting clinical thinking and defining conduct based on the patient’s demands.

The *CuidarTech* ^*®*^ EnfPorElas application, also developed by nurses, consists of a successful experience in nursing care for women in situations of sexual violence^26^. This technology, like IST Nurse^®^, offers a list of diagnoses and interventions that brings representation to nursing care practice. Thus, the importance of artificial intelligence is observed as a tool that assists clinical thinking and decision-making by nurses, contributing to their autonomy and power to solve problems.

The use of AI in healthcare needs to be encouraged and, at the same time, it must be ensured that safe and quality tools are developed, providing user benefit. Therefore, the development of mobile applications based on a reference with technical-scientific support and the analysis of their validity evidence based on the analysis of experienced professionals is necessary^27^.

Mobile services can provide more effective information, serving to offer better knowledge and assist the nurse in conducting a consultation. Furthermore, networks can promote the dissemination of safe guidance on STI, contributing to the reduction of high-risk behaviors^28^.

Despite the availability of clinical protocols and therapeutic guidelines aimed at the clinical management of STI, it is clear that this practice still represents a challenge for health professionals, including nurses. An integrative review carried out based on the analysis of 4 articles and 5 dissertations found deficiencies in the clinical management of syphilis, especially with regard to knowledge about the criteria for diagnosis and treatment, the attitude of inviting sexual partners and practices related to approaching sexual partners, and the prescription/administration of penicillin in basic health units^29^.

Therefore, considering the difficulties presented by health professionals, including nurses, the *IST Nurse* ^*®*^ application was designed to integrate information on the longitudinality of comprehensive care for people with or just suspected STI, even in cases of negative results for rapid tests. On one of its screens, for example, the application presents the combined prevention mandala, guidelines for requesting non-treponemal tests in cases of treponemal tests showing negative results or, also, what the conduct should be in cases in which an STI is suspected even when rapid tests did not confirm the diagnosis.

Even starting from content based on a reference manual recommended by the Brazilian *Ministério da Saúde*, the analysis of validity evidence of content, usability and functional performance by judges is necessary and essential. The experience of the judges who participated in the present study greatly contributed to the identification of inconsistencies between information and/or gaps in relation to the needs of nurses in the clinical management of STI.

The CVI of content (0.98), usability (1.0) and functional performance (0.85) analysis presented acceptable percentages, demonstrating validity evidence for the use of the application by nurses in the clinical management of STI. However, seeking to provide the target audience with the safest and most complete version of the application, all suggestions were analyzed, as they asked about important behaviors in the daily care of people with STI.

Considering the suggestion of one of the judges regarding the immunological window for HIV, when revisiting the *Protocolo Clínico e Diretrizes Terapêuticas* (PCDT)^1^, it was found that there is guidance for requesting a retest after 30 days when there is clinical and/or epidemiological suspicion. In view of the relevance of the subject, this content was added to the application.

One of the judges asked about treatment for syphilis in pregnant women within three weeks. The routine management for this STI considers treating with 2.4 million international units (IU) Benzathine benzylpenicillin, a single dose, in cases of recent syphilis, with some experts recommending an additional dose of 2.4 million IU of benzathine penicillin G one week after the first dose. And, in cases of late syphilis, treat with Benzathine benzylpenicillin 2.4 million IU, once a week, for three weeks^1^.

It is known that health care for STI is mainly concentrated in Primary Health Care (PHC), and that professionals working in this context face challenges in clinical management regarding unpreparedness and lack of knowledge^30^ ^)-(^ ^31^. Applications can be inserted into the practice of care for people with STI, whether in a preventive or diagnostic context, always remembering the importance of notification and treatment^32^ ^)-(^ ^33^.

Considering the notification of STI, the application advises the professional to carry out notification using the health unit’s own form, available in printed form. As the application is in a mobile version, for smartphones, it would not be useful to provide links to access the notification forms, as they are available in a format not capable of being changed.

There was some difficulty in obtaining a larger sample of judges, although invitations were sent to 35 experts to analyze content validity evidence, 30 for experts to participate in the analysis of functional performance validity evidence and 34 for the analysis of usability validity evidence. Many did not return and some responded that they did not have time to collaborate. It should be noted that there was a wait of 45 days before the decision to proceed with the sample presented.

As a limitation, it is noteworthy that the SURE scale presents questions with a negative response meaning, which may bias or induce the participant’s response.

A randomized clinical trial is expected to evaluate the effectiveness of using this application to support STI nursing consultations.

Before the application is commercialized, it is proposed to form a partnership with professional councils to enable professionals to register with the software platform based on their registration. It is believed that in this way the application will be restricted to professionals, thus avoiding self-medication by patients.

It is considered that the product of this study will also be promising for the education of nurses, as it can be used during teaching and extension activities.

Furthermore, the contribution to the practice of nurses working in the context of the FHS is emphasized, where most of these professionals have more contact with the clinical management of STI.

## Conclusion

The content, usability and functional performance of the *IST Nurse* ^*®*^ application presented acceptable validity evidence, meeting the proposal to support the clinical management of STI by nurses.

The focus group technique was relevant in the process of defining requirements, with the *Protocolo Clínico e Diretrizes Terapêuticas para Atenção Integral às Pessoas com Infecções Sexualmente Transmissíveis* being a significant theoretical reference for the construction of the application’s content, as it allowed the creation of information flows based on updated scientific evidence.

The suggestions presented by the judges allowed the improvement of the content and the reduction of any gaps that may arise during the use of the application, considering the possibilities and demands of the care routine.

The technology developed presents good validity evidence, being a proposal to be added to the systematization of assistance that will facilitate the adoption of more coherent conduct in terms of adequate reception, identification and follow-up in the clinical management of STI.
