# Supplementary material for: IST Nurse®: development and validity evidence of a mobile application as a support for the clinical management of Sexually Transmitted Infections
Source: Rev Lat Am Enfermagem. 2024 Sep 2;32:e4317. doi: 10.1590/1518-8345.7207.4317 (PMC11386485; doi:10.1590/1518-8345.7207.4317)
Supplement: Translation

# Translation: *IST Nurse* ^*®*^: elaboração e evidências de validade de aplicativo móvel como subsídio ao manejo clínico de Infecções Sexualmente Transmissíveis*

**DOI:** 10.1590/1518-8345.7207.4318

**Authors:** Leilane Barbosa de Sousa, Ismael Moreira de Sousa, Edmara Chaves Costa, Ana Paula Fragoso de Freitas, Lydia Vieira Freitas dos Santos, Adriana Gomes Nogueira Ferreira

**Affiliations:** 1 Universidade da Integração Internacional da Lusofonia Afro-Brasileira, Redenção, CE, Brasil.; 3 Faculdade Rodolfo Teófilo, Curso de Graduação em Enfermagem, Fortaleza, CE, Brasil.; 4 Universidade Federal do Maranhão, Imperatriz, MA, Brasil.

**Keywords:** Aplicativos Móveis, Tecnologia da Informação, Gerenciamento Clínico, Infecções Sexualmente Transmissíveis, Cuidados de Enfermagem, Saúde Pública

## Abstract

**Objetivo:**

elaborar e avaliar as evidências de validade de um aplicativo móvel para subsidiar o enfermeiro no manejo clínico de infecções sexualmente transmissíveis.

**Método:**

estudo metodológico conduzido em quatro etapas: análise e definição de requisitos; definição do conteúdo; representação computacional com projeto do sistema; e codificação com testes e refinamento. Nas primeiras etapas participaram enfermeiros com expertise na temática, e na última, profissionais com formação em tecnologia da informação e comunicação. A análise dos dados foi realizada por meio do cálculo do Índice de Validade de Conteúdo (IVC), sendo considerado o valor de concordância mínimo de 0,78. Para confirmar a viabilidade do IVC, utilizou-se o teste binomial por meio do *software* R. As variáveis com p > 0,05 indicaram haver concordância entre os juízes.

**Resultados:**

o IVC foi de 0,98 para conteúdo, 1,0 para usabilidade e 0,85 para desempenho funcional, evidenciando que o aplicativo elaborado apresenta alta validade.

**Conclusão:**

o aplicativo *IST Nurse* ^*®*^ representa uma importante ferramenta tecnológica no fortalecimento do cuidado de enfermagem baseado em evidências. Sugere-se, portanto, estudos de intervenção.

## Destaques

**(1)** Inexistência de aplicativos relacionados a IST capazes de subsidiar o manejo clínico.

**(2)** Fortalecimento da APS na identificação e tratamento precoce de IST.

**(3)** Produto útil para atuação clínica, formação e padronização de ações de enfermeiros.

**(4)** Diminuição de falhas na conduta profissional, aumentando a qualidade da assistência.

**(5)** As tecnologias móveis democratizam o acesso à informação, com bom custo-efetividade.

## Introdução

O manejo clínico das infecções sexualmente transmissíveis (IST) ainda se configura como desafio para profissionais de saúde, incluindo enfermeiros. A avaliação clínica ineficaz compromete a quebra da cadeia de transmissão destas infecções, aumentando o número de casos. Nesse ínterim, o raciocínio clínico é um elemento essencial para o processo de tomada de decisão e favorece o diagnóstico e o tratamento precoce, sendo estes fatores essenciais para controle e vigilância das IST^1^ ^)-(^ ^2^.

Detectar e tratar IST de maneira precoce diminui os riscos de propagação, o que ressalta a necessidade de encontrar métodos para melhorar a assistência a pessoas com IST, especialmente no contexto do HIV/Aids^3^. Assim sendo, o enfermeiro pode se beneficiar de ferramentas tecnológicas que possam subsidiar a assistência em enfermagem em ginecologia, urologia e andrologia, contemplando os diversos públicos suscetíveis às IST.

A utilização de tecnologias *eHealth* e seu componente *mHealth* facilita o acesso e a difusão de informações, permitindo a interação e o compartilhamento de conhecimentos entre pessoas em diferentes localizações geográficas^4^. A capacidade de gerar, armazenar e compartilhar informações pertinentes em ritmo acelerado faz dos dispositivos de tecnologia ferramentas atrativas que são cada vez mais utilizadas na rotina de qualquer pessoa que utiliza esses recursos^5^.

Contudo, verifica-se uma lacuna existente na produção e disponibilidade de aplicativos que abordam o manejo clínico das IST. Em revisão narrativa da literatura em janeiro de 2024 para aplicativos móveis que abordam a temática IST, foram encontrados 38 aplicativos para *Android* e três para *iPhone Operating System* (iOS); porém, as tecnologias identificadas não são direcionadas para o manejo clínico de IST.

Acredita-se que um aplicativo móvel elaborado com base em protocolos de atenção integral e em algoritmos que conduzam o enfermeiro ao raciocínio clínico poderá apresentar evidências de validade para subsidiar a assistência de pessoas com sinais e/ou sintomas de IST. Assim, o presente estudo teve como objetivo elaborar e avaliar as evidências de validade de um aplicativo móvel para subsidiar o enfermeiro no manejo clínico de IST.

## Método

### Tipo do estudo

Trata-se de estudo metodológico fundamentado na elaboração e na análise de evidências de validade de aplicativo para dispositivos móveis sobre manejo clínico das IST por enfermeiros. Em síntese, esse tipo de estudo possibilita a inquirição dos métodos de obtenção, sistematização e análise de dados para a construção de um instrumento^6^.

O estudo desenvolveu-se em duas fases. A fase 1 foi a elaboração do aplicativo em quatro etapas: (1) análise e definição de requisitos, (2) definição do conteúdo, (3) representação computacional com projeção do protótipo, e (4) codificação com testes e refinamento. Na fase 2 ocorreu a busca por evidências de validade do aplicativo, em termos de conteúdo, desempenho funcional e usabilidade.

### Local e período do estudo

O estudo foi realizado no município de Redenção, Ceará (CE), Brasil, entre setembro de 2021 e junho de 2022.

### População

Participaram do estudo enfermeiros atuantes na Estratégia de Saúde da Família (ESF) (definição de requisitos para o aplicativo e análise de evidências de usabilidade), enfermeiros que atuam na docência em nível superior (análise de evidências de validade de conteúdo) e profissionais com formação em tecnologia da informação e comunicação (análise de evidências de validade do desempenho funcional).

### Definição de amostra

O convite para participação no estudo ocorreu, inicialmente, via *e-mail*, localizado a partir de pesquisas na Plataforma Lattes do portal do Conselho Nacional de Desenvolvimento Científico e Tecnológico (CNPq). O corpo do *e-mail* ofereceu um resumo contendo título da pesquisa, objetivos e aspectos do método, bem como o Termo de Consentimento Livre e Esclarecido para a prestação de anuência. Essa estratégia, no entanto, não obteve êxito.

Por isso, todos os participantes foram selecionados por amostragem não probabilística, pela técnica de rede de referência^7^. Essa técnica ocorreu a partir da seleção de informantes-chaves denominados sementes, que localizaram algumas pessoas com o perfil necessário para a pesquisa. Essas pessoas indicadas foram solicitadas para indicar novas pessoas com o perfil desejado. A comunicação passou a ser via *WhatsApp,* por onde um vídeo foi enviado com as mesmas informações contidas no *e-mail*.

#### Fase 1: Elaboração do aplicativo

Para a definição dos enfermeiros, foi considerado o método *Design* Centrado no Usuário^8^, no qual é estabelecida a participação/colaboração mútua entre os usuários do aplicativo, sendo considerada eficaz a utilização da técnica de grupo focal nesta fase. Definiu-se a quantidade entre seis a doze participantes por grupo, conforme referência^9^.

Via *WhatsApp*, os possíveis participantes foram contatados e orientados por meio do envio de áudios sobre a proposta do estudo, incluindo questões éticas para participação na pesquisa.

Após 23 dias do início do contato com potenciais participantes da pesquisa, indicados pelos informantes-chave, foi possível selecionar 15 participantes. Diante da incompatibilidade de horários disponíveis para a participação em uma mesma sessão grupal, foi necessário subdividir os profissionais em dois grupos, um constituído por nove enfermeiros e outro por seis.

#### Fase 2: Evidências de validade do aplicativo

Para a seleção dos especialistas para a análise de evidências de desempenho funcional, consideraram-se algumas características definidoras a partir da proposta^10^, que foram adaptadas para este estudo. E para a análise de evidências de usabilidade, foi retomado o contato com os participantes dos dois grupos focais.

Quanto ao número ideal de especialistas, não há consenso na literatura. Assim, foi definido com base nas recomendações^7^, que propõem a quantidade mínima de seis especialistas. Além dessa recomendação, é aconselhado usar um número ímpar de especialista para evitar empate de opiniões^7^. Essa recomendação foi adotada para a análise de evidências de conteúdo e de usabilidade. Pela dificuldade em encontrar especialistas disponíveis para a análise de evidências de desempenho funcional, e a partir de referência específica para a avaliação de *software*, encontrou-se que essa análise ocorre bem com o número de três a cinco especialistas^11^.

Foram selecionados sete especialistas para a evidência de validade de conteúdo, três para a de desempenho funcional, e sete para a análise de evidências de usabilidade.

### Critérios de seleção

#### Fase 1: Elaboração do aplicativo

Foram incluídos enfermeiros atuantes na ESF com pelo menos um ano de experiência, uma vez que, a partir do trabalho cotidiano com a pessoa/família/coletividade, possuem experiência na assistência a pessoas com IST. A determinação temporal relaciona-se à percepção dos autores sobre um período mínimo para que a vivência na prática do manejo clínico permita propriedade suficiente para apontar as potencialidades e as fragilidades de sua atuação nesse campo. Foram excluídos os que estavam de férias ou afastados por algum tipo de licença.

#### Fase 2: Evidências de validade do aplicativo

Foram considerados especialistas para a análise de evidências de conteúdo enfermeiros pesquisadores/docentes com experiência na área de interesse: saúde sexual, IST e/ou tecnologias em saúde. Foram excluídos os que não atingiram pelo menos cinco pontos pela aplicação dos critérios adaptados^10^, sendo estes: ser doutor ou mestre com *expertise* na área de interesse.

Para a análise de evidências de validade de usabilidade participaram como especialistas os enfermeiros que colaboraram na etapa de definição de requisitos, na fase 1 do estudo. Os critérios de inclusão e de exclusão já foram explicados e justificados anteriormente.

Para a análise de evidências de desempenho funcional foram incluídos profissionais com formação e experiência nas áreas de engenharia de *softwares*, ciências da computação e análise e desenvolvimento de sistemas, e excluídos os que não atenderam aos critérios adaptados^10^, sendo estes: diploma de graduação em Tecnologia da Informação e Comunicação, experiência profissional em tecnologia da informação/comunicação/computação pelo período mínimo de dois anos e experiência no desenvolvimento e implantação de sistemas.

### Fases do estudo

#### Fase 1: Elaboração do aplicativo

Seguiram-se quatro etapas utilizando método incremental e interativo: (1) análise e definição de requisitos; (2) definição do conteúdo; (3) representação computacional com projeto do sistema; e (4) codificação com testes e refinamento^11^ ^)-(^ ^14^.

Na análise e definição de requisitos, buscou-se compreender principalmente o conhecimento e a atuação dos enfermeiros com relação ao manejo clínico das IST. Essa etapa permitiu acessar as demandas dos enfermeiros sobre o que o aplicativo precisaria ofertar.

Após a identificação das demandas dos enfermeiros, buscou-se parceria com uma equipe com experiência em análise e desenvolvimento de *softwares*, liderada por um docente pesquisador Doutor em Informática, da Universidade Federal do Maranhão. A equipe e um dos autores da proposta realizaram seis reuniões para definir as funcionalidades desejadas para o aplicativo e fazer o levantamento de requisitos para, posteriormente, utilizar a técnica de prototipação^15^. O mesmo autor foi qualificado pela equipe parceira para desenvolver a primeira versão do protótipo do aplicativo por meio da plataforma Figma^16^, na qual foram definidos aspectos básicos e funcionais do sistema, como fluxos de interação, localização do botão, texto e mapeamento de imagens.

A etapa de definição de conteúdo objetivou reunir as melhores evidências científicas sobre o tema proposto, e optou-se por utilizar como referência base dois protocolos do Ministério da Saúde^1^ ^),(^ ^17^. Estes são os referenciais brasileiros mais atuais sobre a temática.

A etapa de representação computacional com projeto do sistema consistiu na formatação do conteúdo em um molde adequado para posterior codificação em Java. Isso resultou na versão 1 do protótipo, que incorporou o corpo textual formulado a partir da análise e definição de requisitos, seguindo fluxos lógicos de funcionamento. A logo do aplicativo foi criada usando o *software* Canva^18^.

Após a avaliação da versão 1 pela equipe de desenvolvimento, a versão 2 foi estruturada com base em projetos disponibilizados pela comunidade do Figma^16^, seguindo padrões de *design* e Interface Humano-Computador (IHC).

Na etapa de codificação com testes e refinamento, o aplicativo foi desenvolvido em módulos, permitindo testes de qualidade à medida que cada módulo foi implementado. Isso facilitou a detecção precoce de falhas para correções rápidas.

Para a codificação, foi utilizada a linguagem de programação *Javascript* com o *framework React Native*, permitindo a criação de aplicativos para *Android* e *iOS*. O controle de versões foi realizado com as ferramentas *Git* e *Github*, e a instalação de pacotes foi realizada com npm e yarn.

Após a codificação, o aplicativo foi instalado em um servidor *Expo* em nuvem e foram gerados um *link* e um *Quick Response Code* para acesso. Isso permitiu que os pesquisadores e especialistas envolvidos no estudo acessassem o aplicativo para testes e refinamentos.

#### Fase 2: Evidências de validade do aplicativo

Ocorreu em três campos: conteúdo, desempenho funcional e usabilidade. Para a definição dos especialistas de conteúdo, foi adaptado um conjunto de requisitos propostos^10^.

### Instrumentos utilizados e coleta de dados

#### Fase 1: Elaboração do aplicativo

As sessões de grupo focal ocorreram na modalidade remota e síncrona, e foram gravadas por *e-mail* institucional de um dos autores para posterior resgate das falas. Foram utilizadas questões disparadoras durante as reuniões por um autor que moderou o momento, o que viabilizou a discussão grupal.

O material gravado foi revisitado para apreciação, o que possibilitou a definição das necessidades dos profissionais e a definição do conteúdo que deveria compor o aplicativo.

Identificadas as demandas dos enfermeiros no manejo clínico das IST, buscou-se o conteúdo pertinente em manual e protocolo disponibilizados pelo Ministério da Saúde sobre a temática^1^ ^),(^ ^17^.

#### Fase 2: Evidências de validade do aplicativo

Para a evidência de validade de conteúdo foi utilizado um instrumento adaptado^19^, que contempla itens distribuídos nos seguintes blocos: objetivos e conteúdo (propósitos e metas ou fins que se deseja atingir por meio da prática com a tecnologia) e estrutura e funcionalidade (forma de apresentar as orientações e praticidade nas funções).

No processo de análise de evidências de validade de desempenho funcional foi utilizado um instrumento fundamentado no Modelo de Qualidade de Produto da norma *International Organization for Standardization*/*International Electrotechnical Commission* (ISO/IEC) 25010, que especifica oito características de qualidade^20^.

Para a análise de evidências de validade de usabilidade foi utilizada a escala *Smartphone Usability Questionnaire* (SURE), com o objetivo de possibilitar que o usuário final avaliasse o aplicativo de forma subjetiva diretamente observada durante o teste^21^.

Para cada item dos instrumentos e critério avaliado foi atribuída nota 1 - inadequado, 2 - parcialmente adequado, 3 - adequado, 4 - totalmente adequado e 5 - não se aplica.

Os especialistas participantes das três modalidades de processo de análise de evidências de validade receberam os instrumentos em formulário via *e-mail* e via mensagem por *WhatsApp*. Nos instrumentos foram disponibilizados espaços para que os especialistas realizassem comentários e sugestões sobre cada item avaliado. Decorridos 14 dias, as respostas de todos estavam disponíveis.

### Tratamento e análise dos dados

Foi realizada uma reunião remota síncrona com cada grupo focal. Os detalhes do estudo foram apresentados e as questões disparadoras lançadas. As falas registradas pela gravação da voz e textos foram transcritas e processadas. Os discursos gerados pelas reuniões com os grupos focais foram analisados seguindo o método do discurso do sujeito coletivo^22^. Como ferramenta de auxílio nessa análise, lançou-se mão do *software* IRaMuTeq.

No processo de análise, foi utilizado o Índice de Validade de Conteúdo (IVC), a fim de mensurar o percentual de concordância para cada item individualmente. O IVC foi calculado pela soma dos itens 3 (adequado) e 4 (inadequado) dividido pelo número total de respostas, sendo considerado o valor de concordância mínimo 0,78^23^. No caso de uma concordância menor, o item seria modificado de acordo com sugestões apontadas pelos especialistas.

Para confirmar a viabilidade do IVC, foi utilizado, ainda, o teste binomial a partir do processamento do comando binom.test (n° de sucessos, n° total, p=05) no *software* R: aqui foi testado se a proporção de sucesso observada na amostra pertencia a uma população com um determinado valor de *p*, em que valores de *p* superiores a 0,05 indicavam haver concordância entre os especialistas, não sendo estatisticamente inferior a 85%^24^.

### Aspectos éticos

A pesquisa foi aprovada pelo Comitê de Ética e Pesquisa da Universidade da Integração Internacional da Lusofonia Afro-Brasileira sob parecer de número 4.889.214.

## Resultados

Foram geradas pelo IRaMuTeq uma nuvem de palavras e uma árvore de similitude e, a partir da apreciação destas, formaram-se categorias temáticas seguindo o método de análise do discurso do sujeito coletivo. Essas categorias foram importantes para o processo de definição do conteúdo.

Verificou-se, por meio dos grupos focais, que as classes mais relevantes para o conteúdo do aplicativo são: dificuldades relacionadas ao conhecimento do profissional sobre o manejo, reconhecimento de sintomas e falta de adesão ao tratamento por parte das parcerias sexuais, uso dos protocolos do Ministério da Saúde como consulta para manejo clínico, e a praticidade e a facilidade oferecidas por tecnologias como aplicativos móveis para orientar o tratamento adequado para IST.

Essas classes foram a base para a esquematização do conteúdo teórico do aplicativo, tendo como principais referências o Protocolo Clínico e Diretrizes Terapêuticas para Atenção Integral às Pessoas com IST^1^ e o Caderno de Atenção Básica 18: HIV/Aids, Hepatites Virais, Sífilis e outras IST^17^, ambos do Ministério da Saúde.

Seguindo o padrão *React Native*, foram construídas 121 telas, organizadas em subcategorias de pastas conforme as IST e seções do aplicativo. Por exemplo, dentro da pasta de “Tira Dúvidas” existem subpastas referentes a cada infecção. Essa estrutura foi definida para facilitar a organização dos arquivos que compõem o sistema, além de permitir a sua posterior manutenção.

O aplicativo não se utiliza de nenhum *back-end* para processamento de dados, tratando- se somente de um aplicativo de exposição de informações e navegações. Deste modo, todas as telas desenvolvidas são estáticas, apresentando informações relativas às IST e a possibilidade de navegação (voltando ou avançado nas telas). Além das telas referentes ao manejo clínico de IST, foram desenvolvidas outras sobre os idealizadores do projeto, desenvolvedores e referências.

Após todo o processo de desenvolvimento, no total foram escritas aproximadamente 10.000 linhas de código, sendo utilizadas 54 imagens. Então, o cliente com o aplicativo do Expo - para testes e refinamento - pode, através do *link* ou *Quick Response Code*, abrir o sistema da nuvem em seu celular. Isso foi realizado pela necessidade de executar o sistema em um celular com sistema operacional *Android* e *iOS*, e esse sistema operacional não possuir suporte e pacotes de instalação.

Assim como foi projetado na prototipação, o aplicativo segue um fluxo que foi codificado nessa fase. Esse fluxo obedece a comandos definidos a partir do conteúdo utilizado, com uma inteligência computacional aplicada ao processo de manejo clínico das IST.

A versão codificada do sistema, realizada através da linguagem de programação *Javascript* utilizada pelo *framework React Native*, apresenta um aplicativo interativo, seguindo o fluxo descrito anteriormente, sendo de fácil uso e navegação, contendo informações textuais e imagens, com base científica, fundo da tela escuro com textos em cor branca e comandos na cor verde.

As telas iniciais do aplicativo estão apresentadas na Figura 1, que mostra o percurso da tela *Home* até a conduta terapêutica a partir do manejo clínico.

**Figura 1:** Fluxo de passos do aplicativo da tela *home* até o direcionamento ao tratamento indicado para um corrimento vaginal. Redenção, CE, Brasil, 2022

### Análise das evidências de validade de conteúdo

Os sete especialistas enfermeiros e com experiência na docência em nível superior, assim como todos, participaram de grupo de pesquisa. As áreas de atuação foram saúde sexual e reprodutiva (n=2), saúde da família (n=3) e saúde coletiva (n=2), e a média de tempo de atuação nas áreas foi de 8,43 anos. Todos obtiveram pontuação superior a cinco, concernente ao somatório dos escores atribuídos pelo conjunto de requisitos.

Quatro (57,2%) especialistas eram mestres, e três (42,9%) doutores. Um dos especialistas orientou um aluno de mestrado, quatro orientaram alunos para elaboração de monografia ou trabalho de conclusão de curso. Todos tinham publicações envolvendo temáticas de interesse. Três especialistas conciliavam atuação entre ensino e pesquisa, e três tinham pesquisas publicadas envolvendo as temáticas IST e Tecnologia em Saúde.

De acordo com a Tabela 1, a avaliação dos especialistas evidenciou uma média 4 (totalmente adequado) para a grande parte dos itens avaliados, havendo uma média de 3,71 para o item que avalia a organização das informações. Sendo assim, os itens apresentaram IVC com valores estatisticamente significantes, com uma proporção de concordância entre os especialistas superior a 85% (*p*>0,05).

**Tabela 1:** Distribuição das respostas dos juízes sobre o conteúdo apresentado pelo aplicativo IST* *Nurse* ^*®*^ . Redenção, CE, Brasil, 2022

N°	Questões	mín^†^	méd^‡^	máx^§^	desv pd^||^	IVC^¶^	p**
1	O aplicativo mantém coerência com as necessidades dos enfermeiros no que tange ao raciocínio clínico para o manejo das IST*?	4	4	4	0	1	1
2	O aplicativo promove a sistematização para o manejo clínico das IST*?	4	4	4	0	1	1
3	O aplicativo pode circular no meio científico na área do manejo clínico de IST*?	4	4	4	0	1	1
4	O material do aplicativo é adequado para o subsídio do enfermeiro durante o manejo clínico das IST*?	4	4	4	0	1	1
5	As informações estão organizadas de forma clara e objetiva?	3	3.71	4	0.49	1	1
6	As informações apresentadas estão coerentes com as evidências científicas?	4	4	4	0	1	1
7	Há uma sequência lógica do conteúdo proposto?	4	4	4	0	1	1
8	O material está adequado quanto ao nível sociocultural do público-alvo proposto?	4	4	4	0	1	1
9	As informações são bem estruturadas em concordância e ortografia?	2	3.29	4	0.95	0,76	0,71
10	O estilo de redação corresponde ao nível de conhecimento do público-alvo proposto?	3	3.86	4	0.38	1	1
11	As ilustrações são expressivas e suficientes?	3	3.43	4	0.53	1	1
12	O número de passos está adequado?	3	3.86	4	0.38	1	1
13	O tamanho dos itens e tópicos estão adequados?	3	3.86	4	0.38	1	1
14	Os temas retratam os aspectos chave que devem ser atentados?	3	3.86	4	0.38	1	1
15	O aplicativo oferece aos enfermeiros os requisitos suficientes direcionados às pessoas com IST* para o manejo clínico das IST*?	3	3.86	4	0.38	1	1
16	O aplicativo está adequado para ser utilizado por qualquer enfermeiro que atue na atenção às pessoas com IST*?	3	3.86	4	0.38	1	1

*IST = Infecção sexualmente transmissível; ^†^mín = Pontuação mínima; ^‡^méd = Média; ^§^máx = Pontuação máxima; ^||^desv pd = Desvio-padrão; ^¶^IVC = Índice de validade de conteúdo; **p = Teste binomial

O único item que apresentou baixa proporção foi o nove, que remete à concordância e à ortografia do conteúdo, que recebeu média 3,29. As considerações apontadas pelos especialistas foram atendidas por meio de revisão e correção ortográficas.

Os erros no aplicativo foram atribuídos a equívocos de digitação e ao não reconhecimento de certos termos pela plataforma, resultando na geração automática de palavras próximas. Os especialistas elogiaram a síntese e a completude do conteúdo, a sistematização e a agilidade do aplicativo, mas sugeriram mais ilustrações para melhorar o manejo clínico. Eles também destacaram a eficiência do aplicativo em abordar os aspectos-chave do conteúdo, com sugestões para incluir informações sobre hepatites e a janela imunológica.

O ajuste do aplicativo foi realizado com base nesses comentários. Com um desvio-padrão indicando homogeneidade na amostra e um Índice de Validade de Conteúdo (IVC) de 0,98, o aplicativo mostra evidências de validade e é considerado útil para enfermeiros que lidam com IST.

### Análise das evidências de validade da usabilidade

Participaram dessa etapa sete especialistas, todos enfermeiros formados entre 2015 e 2020, com uma média de experiência de 3,29 anos na ESF. A maioria (85,71%) era do sexo feminino, enquanto apenas um era do sexo masculino (14,29%). Seis eram especialistas (85,71%) e um era mestre (14,29%).

A avaliação do aplicativo seguiu a escala SURE^21^ para a análise de evidências de validade de usabilidade. As respostas dos especialistas indicaram que algumas questões foram consideradas positivas na prática, mas discordaram das afirmações propostas pela escala, não sendo utilizadas para o cálculo do IVC.

De acordo com a Tabela 2, as médias de avaliação das questões da escala anteriormente citada variaram entre 3,57 e 3,86, mostrando homogeneidade na amostra quanto à adequabilidade do aplicativo para o manejo clínico de IST. O IVC foi de 1,0, refletindo unanimidade na avaliação positiva do aplicativo pelos enfermeiros. As falas dos especialistas corroboraram os dados, mostrando satisfação com a aparência, usabilidade e conteúdo teórico do aplicativo, destacando sua praticidade e capacidade de facilitar a prática dos enfermeiros com pessoas com IST. 

**Tabela 2:** Distribuição das respostas dos especialistas de usabilidade do aplicativo IST* *Nurse* ^*®*^ . Redenção, CE, Brasil, 2022

N°	Questões	mín^†^	méd^‡^	máx^§^	desv pd^||^	IVC^¶^	p^**^
1	Eu achei fácil inserir dados neste aplicativo. Por exemplo, utilizando código QR, lista de opções, etc.	3	3.57	4	0.53	1	1
2	Quando eu cometo um erro, é fácil de corrigi-lo.	3	3.57	4	0.53	1	1
3	Eu achei que a ajuda/dica dada pelo aplicativo foi útil.	3	3.71	4	0.49	1	1
4	Foi fácil encontrar as informações que precisei.	3	3.71	4	0.49	1	1
5	Eu me senti no comando usando este aplicativo.	3	3.71	4	0.49	1	1
6	Eu achei adequado o tempo que levei para completar as tarefas.	3	3.71	4	0.49	1	1
7	Foi fácil de aprender a usar este aplicativo. (Para responder essa questão, você deve considerar apenas o uso do aplicativo IST* *Nurse* ^*®*^ , e não o processo de instalação).	3	3.71	4	0.49	1	1
8	A sequência das ações no aplicativo corresponde à maneira como eu normalmente as executo, por exemplo, a ordem de botões, campos de dados, etc.	3	3.57	4	0.53	1	1
9	É fácil de fazer o que eu quero usando este aplicativo.	3	3.71	4	0.49	1	1
10	Foi fácil navegar nos menus e telas do aplicativo.	3	3.86	4	0.38	1	1
11	O aplicativo atende às minhas necessidades.	3	3.86	4	0.38	1	1
12	Eu recomendaria este aplicativo para outras pessoas.	3	3.86	4	0.38	1	1
13	Mesmo com pressa eu conseguiria executar as tarefas nesse aplicativo.	3	3.86	4	0.38	1	1
14	Eu achei o aplicativo consistente. Por exemplo, todas as funções podem ser realizadas de uma maneira semelhante.	3	3.71	4	0.49	1	1
15	É fácil lembrar como fazer as coisas neste aplicativo.	3	3.86	4	0.38	1	1
16	Eu usaria este aplicativo com frequência.	3	3.71	4	0.49	1	1
17	A organização dos menus e comandos de ação (como botões e *links*) é lógica, permitindo encontrá-los facilmente na tela.	3	3.86	4	0.38	1	1
18	Eu consegui completar as tarefas com sucesso usando este aplicativo.	3	3.86	4	0.38	1	1
19	Eu gostei de usar este aplicativo.	3	3.86	4	0.38	1	1
20	O aplicativo oferece todas as informações necessárias para completar as tarefas de forma clara e compreensível.	3	3.86	4	0.38	1	1
21	Eu achei o aplicativo muito complicado de usar.	1	1	1	0	0	0
22	Os símbolos e itens são claros e intuitivos.	3	3.71	4	0.49	1	1
23	Eu achei os textos fáceis de ler.	3	3.86	4	0.38	1	1
24	Eu achei o aplicativo desnecessariamente complexo. Precisei lembrar, pesquisar ou pensar muito para completar as tarefas.	1	1	1	0	0	0
25	A terminologia utilizada nos textos, rótulos, títulos, etc., é fácil de entender.	3	3.71	4	0.49	1	1
26	Eu precisaria do apoio de uma pessoa para usar este aplicativo.	1	1.29	2	0.49	0	0
27	Eu me senti confortável usando este aplicativo.	3	3.71	4	0.49	1	1
28	O aplicativo se comportou como eu esperava.	3	3.71	4	0.49	1	1
29	Eu achei frustrante usar este aplicativo.	1	1	1	0	0	0
30	Eu achei que as várias funções do aplicativo são bem integradas.	3	3.57	4	0.53	1	1
31	Eu me senti muito confiante usando este aplicativo.	3	3.86	4	0.38	1	1

*IST = Infecção sexualmente transmissível; ^†^mín = Pontuação mínima; ^‡^méd = Média; ^§^máx = Pontuação máxima; ^||^desv pd = Desvio-padrão; ^¶^IVC = Índice de validade de conteúdo; **p = Teste binomial

### Análise das evidências de validade do desempenho funcional

Participaram dessa etapa três especialistas, com formação em Ciências da Computação (n=2) e Sistemas de Informação (n=1), sendo este último do sexo feminino. O ano de formação está entre 2015 e 2021, tendo como média 3,67 anos de experiência na área de formação.

A Tabela 3 apresenta o resultado da avaliação dos especialistas que realizaram a análise das evidências de validade do desempenho funcional. As médias da avaliação das questões ficaram entre 3 e 4, o que indica que os especialistas concordaram que as funcionalidades do aplicativo estão adequadas ou totalmente adequadas. O desvio-padrão corroborou isso, variando de 0 a 0,58. O IVC foi significante estatisticamente, e, além disso, o valor de *p* para todos os itens evidencia proporção de concordância de 100% entre os especialistas.

**Tabela 3:** Distribuição das respostas dos especialistas de desempenho funcional do aplicativo IST* *Nurse* ^*®*^ . Redenção, CE, Brasil, 2022

N°	Questões	mín^†^	méd^‡^	máx^§^	desv pd^||^	IVC^¶^	p^**^
1	As funções disponíveis são suficientes para realizar as tarefas que o aplicativo propõe?	4	4	4	0	1	1
2	A linguagem utilizada no aplicativo apresenta-se de forma compreensível?	3	3.67	4	0.58	1	1
3	O aplicativo está condizente com o público-alvo a que se destina?	4	4	4	0	1	1
4	A quantidade de informações colocadas em cada tela é apropriada ao público-alvo?	3	3.67	4	0.58	1	1
5	As cores são utilizadas com equilíbrio, ou seja, são bem distribuídas, evitando assim a poluição visual?	3	3.67	4	0.58	1	1
6	Com o uso do aplicativo, é possível obter informações para alcançar seu objetivo primordial?	3	3.67	4	0.58	1	1
7	Convida ou instiga o usuário ao raciocínio sistemático?	4	4	4	0	1	1
8	As informações contidas no aplicativo estão coerentes?	4	4	4	0	1	1
9	É atrativo ao usuário?	4	4	4	0	1	1
10	É fácil entender os conceitos utilizados?	4	4	4	0	1	1
11	As funções são fáceis de aprender a usar?	4	4	4	0	1	1
12	As apresentações das funções da interface (ícones, menus...) são fáceis de serem entendidas?	3	3.67	4	0.58	1	1
13	É fácil operar e controlar a operação?	4	4	4	0	1	1
14	O aplicativo possui consistência com o que se espera em relação ao proposto cientificamente?	4	4	4	0	1	1
15	Na presença de erros, o aplicativo permite recuperação dos dados já fornecidos?	3	3	3	NA^††^	1	1
16	Evita acesso não autorizado, acidental ou deliberado a programas de dados?	3	3.5	4	0.71	1	1
17	Promove integridade do usuário?	4	4	4	0	1	1
18	É fácil encontrar falhas quando ocorrem?	3	3	3	NA^††^	1	1
19	É fácil adaptar a outros ambientes sem aplicar outras ações ou meios além dos fornecidos para esta finalidade no *software* considerado?	3	3.67	4	0.58	1	1
20	O aplicativo é fácil de ser instalado?	3	3.67	4	0.58	1	1
21	Tem boa capacidade de ser substituído?	4	4	4	NA^††^	1	1
22	Emite alguma forma de *feedback* quando o usuário segue uma linha negativa de raciocínio?	4	4	4	0	1	1
23	Oferece resumo do desempenho do usuário ao final de sua utilização?	4	4	4	0	1	1

*IST = Infecção sexualmente transmissível; ^†^mín = Pontuação mínima; ^‡^méd = Média; ^§^máx = Pontuação máxima; ^||^desv pd = Desvio-padrão; ^¶^IVC = Índice de validade de conteúdo; **p = Teste binomial; ^††^NA = Não se aplica

Quanto as questões 15 e 17, tendo em vista que um dos especialistas apontou a necessidade de cadastro do paciente no aplicativo, trata-se de algo que, por decisão dos pesquisadores, foi desconsiderado em virtude da proteção dos dados do paciente, uma vez que o aplicativo estará em uso no celular do enfermeiro, e não de um aparelho exclusivo do serviço. O aplicativo objetiva conduzir o manejo clínico de IST no que diz respeito às características propedêuticas evidenciadas durante a consulta de enfermagem, as quais serão subsídio para o pensamento crítico e reflexivo sobre a avaliação clínica. Contudo, os demais dados pessoais deverão ser considerados pelo profissional para registro em documentos próprios do serviço.

Em relação às questões 16 e 23, as ponderações foram levadas à equipe que desenvolveu o sistema para incrementar uma forma de acesso restrito ao profissional que tiver o aplicativo *IST Nurse* ^*®*^ em seu *smartphone*, assim como oferecer um resumo dos passos seguidos pelo usuário e a conduta adotada.

Das questões 15 a 18, e 21 a 23, alguns especialistas marcaram a opção “NA” para não se aplica. Isso traz a informação de que o avaliador não gostaria de responder àquela questão por considerá-la inadequada para o objetivo do processo avaliativo. Tendo em vista isso, nas questões em que havia essa opção marcada por algum especialista, este não foi considerado como respondente para o cálculo do IVC.

O IVC apresentou valor 0,85, representando ótimo nível de concordância entre os especialistas, e reflete que o aplicativo tem comandos e funções adequados ou totalmente adequados para cumprir o que se propõe.

Ao final da exposição desses dados, apresenta-se a Figura 2, que mostra as sugestões dos especialistas para aprimoramento do aplicativo, bem como aponta se as sugestões foram ou não acatadas pelos autores, e de que forma.

**Figura 2:** Sugestões dos juízes e decisão dos autores sobre mudanças no primeiro molde do aplicativo. Redenção, CE, Brasil, 2022

Sugestão	Decisão
**Análise de evidências de conteúdo**	
Ilustrar melhor a tela inicial.	Acatada. A logo foi modificada para propor maior relação com o objetivo do aplicativo.
Inserir mais imagens para auxiliar o enfermeiro a melhor identificar/suspeitar durante o manejo.	Acatada. Novas imagens foram inseridas para a clínica apresentada das infecções.
Melhorar a qualidade das imagens.	
Aprimorar as informações sobre a conduta no manejo da sífilis, especialmente a respeito da cicatriz sorológica.	Acatada. Foi acrescentado conteúdo para orientação ao enfermeiro quanto à necessidade de solicitação de exames e conduta para o acompanhamento dos casos de sífilis.
Abordar a janela imunológica na questão dos testes rápidos.	Acatada. Foi inserida a informação para solicitação de nova coleta em 30 dias em caso de suspeita ou resultados negativos.
Melhorar as imagens dos testes rápidos.	Acatada. Foram inseridas novas imagens para auxiliar na interpretação dos resultados dos testes rápidos.
Inserir o tratamento para as verrugas anogenitais.	Acatada. Foi inserida a indicação de tratamento.
**Análise de evidências do desempenho funcional**	
Facilitar o processo de instalação inserindo em uma das lojas.	Não acatada. O aplicativo só será disponibilizado nas lojas após aplicação em estudo de avaliação de efetividade.
**Análise de evidências de usabilidade**	
Inserir um *link* no aplicativo que direcione para a notificação.	Não acatada. Essa possibilidade havia sido pensada na prototipação, mas por orientação da equipe de desenvolvimento do *software* não foi inserido.

## Discussão

A elaboração de uma tecnologia direcionada para o cotidiano de enfermeiros precisa considerar a demanda que estes apresentam. Durante a elaboração do aplicativo *IST Nurse* ^*®*^ , a estratégia de grupo focal *online* mostrou-se eficaz na identificação de temas que deveriam compor o conteúdo da ferramenta tecnológica, uma vez que permitiu o diálogo com profissionais que atuam em diferentes cidades e a verificação de necessidades e prioridades no âmbito do manejo clínico de IST. Estudo semelhante, que também utilizou o grupo focal *online* como estratégia de diagnóstico situacional, ressaltou que esta estratégia contribuiu de forma significativa na construção de conhecimentos na área da saúde^25^.

As demandas elencadas a partir do diálogo com os enfermeiros constituíram o ponto de partida para a produção do conteúdo do aplicativo. Nesse percurso, o Protocolo Clínico e Diretrizes Terapêuticas para Atenção Integral às Pessoas com Infecções Sexualmente Transmissíveis (PCDT-IST)^1^ configurou-se como relevante referência para o embasamento do fluxo dos dados no aplicativo, uma vez que apresenta informações fundamentadas em evidências científicas sobre as características das IST, e critérios para diagnósticos e condutas, incluindo não apenas tratamento farmacológico, mas também orientações voltadas à prevenção e controle, essenciais ao cuidado de enfermagem. A partir do PCDT-IST foi possível elaborar conteúdo direcionado à condução do pensamento clínico e da definição de condutas a partir das demandas do paciente.

O aplicativo *CuidarTech* ^*®*^ EnfPorElas, também desenvolvido por enfermeiros, consiste em experiência exitosa na assistência de enfermagem a mulheres em situação de violência sexual^26^. Essa tecnologia, assim como o *IST Nurse* ^*®*^ , oferece uma lista de diagnósticos e intervenções que traz representatividade para a prática assistencial de enfermagem. Observa-se, assim, a importância da inteligência artificial como ferramenta que auxilia o pensamento clínico e a tomada de decisão pelo enfermeiro, contribuindo para sua autonomia e poder de resolubilidade.

O uso da IA na saúde precisa ser estimulado e, concomitantemente, deve ser assegurado que sejam desenvolvidas ferramentas seguras e de qualidade, propiciando o benefício do usuário. Assim, a elaboração de aplicativos móveis a partir de referencial com respaldo técnico-científico e a análise de suas evidências de validade a partir da análise de profissionais com experiência se faz necessária^27^.

Os serviços móveis podem fornecer mais informações efetivas, servindo para oferecer melhor conhecimento e auxiliar o enfermeiro a conduzir uma consulta. Além disso, as redes podem favorecer a disseminação de orientações seguras sobre IST, contribuindo para a redução de comportamentos de alto risco^28^.

Apesar da disponibilização de protocolos clínicos e de diretrizes terapêuticas direcionadas ao manejo clínico de IST, percebe-se que esta prática ainda se configura como um desafio para profissionais de saúde, incluindo enfermeiros. Revisão integrativa realizada a partir da análise de quatro artigos e cinco dissertações verificou deficiências no manejo clínico da sífilis, especialmente no que concerne ao conhecimento sobre os critérios para diagnóstico e tratamento, atitude de convocar parcerias sexuais e práticas relacionadas à abordagem de parcerias sexuais, e prescrição/administração de penicilina nas unidades básicas de saúde^29^.

Sendo assim, pensando nas dificuldades apresentadas por profissionais de saúde, incluindo enfermeiros, o aplicativo *IST Nurse* ^*®*^ foi elaborado para integrar informações sobre a longitudinalidade da atenção integral às pessoas com IST ou apenas com suspeita, mesmo em casos de negatividades para os testes rápidos. Em uma de suas telas, por exemplo, o aplicativo apresenta a mandala da prevenção combinada, orientações para a solicitação de testes não-treponêmicos em casos de testes treponêmicos apresentando negatividade ou, ainda, quais as condutas em casos em que há suspeita de IST mesmo quando os testes rápidos não confirmaram o diagnóstico.

Mesmo partindo de um conteúdo baseado em um manual de referência recomendado pelo Ministério da Saúde do Brasil, a análise de evidências de validade de conteúdo, de usabilidade e de desempenho funcional por especialistas é necessária e imprescindível. A experiência dos especialistas que participaram do presente estudo contribuiu sobremaneira para a identificação de inconsistências entre informações e/ou lacunas com relação às necessidades do enfermeiro no manejo clínico das IST.

O IVC das análises de conteúdo (0,98), da usabilidade (1,0) e do desempenho funcional (0,85) apresentou percentuais aceitáveis, mostrando evidências de validade para o uso do aplicativo por enfermeiros no manejo clínico de IST. Contudo, buscando fornecer ao público-alvo a versão mais segura e completa do aplicativo, todas as sugestões foram analisadas, uma vez que indagavam sobre condutas importantes no cotidiano da assistência às pessoas com IST.

Considerando a sugestão de um dos especialistas sobre a janela imunológica para HIV, ao revisitar o Protocolo Clínico e Diretrizes Terapêuticas (PCDT)^1^, constatou-se a orientação para a solicitação de reteste após 30 dias quando houver suspeita clínica e/ou epidemiológica. Em vistas da pertinência do assunto, esse conteúdo foi acrescido ao aplicativo.

Um dos especialistas indagou sobre o tratamento para sífilis em gestantes ser em três semanas. A rotina do manejo para essa IST considera tratar com Benzilpenicilina benzatina 2,4 milhões unidades internacionais (UI), dose única, em casos de sífilis recente, sendo que alguns médicos especialistas recomendam uma dose adicional de 2,4 milhões de UI de penicilina G benzatina uma semana após a primeira dose. E, em casos de sífilis tardia, tratar com Benzilpenicilina benzatina 2,4 milhões UI, uma vez por semana, por três semanas^1^.

Sabe-se que a assistência à saúde para IST é concentrada principalmente na Atenção Primária à Saúde (APS), e que os profissionais atuantes nesse contexto enfrentam desafios para o manejo clínico concernentes ao despreparo e à falta de conhecimento^30^ ^)-(^ ^31^. Os aplicativos podem ser inseridos na prática do cuidado a pessoas com IST, seja em âmbito preventivo ou para diagnóstico, lembrando sempre da importância da notificação e do tratamento^32^ ^)-(^ ^33^.

Pensando na notificação das IST, o aplicativo orienta que o profissional realize notificação com ficha própria da unidade de saúde, disponibilizadas impressas. Como o aplicativo está em uma versão mobile, para *smartphones*, não teria aproveitamento a disponibilização de *links* para acesso às fichas de notificação, uma vez que elas estão disponíveis em formato não habilitado para alteração.

Houve certa dificuldade para obter uma amostra maior de especialistas, apesar de terem sido enviados convites para 35 especialistas para a análise de evidências de validade de conteúdo, 30 para especialistas participarem da análise de evidências de validade de desempenho funcional e 34 para a análise de evidências de validade de usabilidade. Muitos não retornaram e alguns responderam que não estavam com tempo hábil para colaborar. Ressalta-se que houve uma espera de 45 dias até a decisão de seguir com a amostra apresentada.

Como limitação, destaca-se que a escala SURE apresenta questões com sentido de resposta negativo, o que pode enviesar ou induzir a resposta do participante.

Especula-se um ensaio clínico randomizado para avaliar a efetividade do uso deste aplicativo como apoio às consultas de Enfermagem em IST.

Antes da comercialização do aplicativo, propõe-se a parceria com os conselhos de classe para um possível cadastro do profissional à plataforma do *software* a partir do seu registro. Acredita-se que dessa forma o aplicativo ficará restrito aos profissionais, evitando assim a automedicação por parte dos pacientes.

Considera-se que o produto desse estudo será promissor também para a formação dos enfermeiros, uma vez que poderá ser empregado durante as atividades de ensino e extensão.

Além disso, enfatiza-se a contribuição para a prática dos enfermeiros atuantes no contexto na ESF, local onde majoritariamente esse profissional tem mais contato com o manejo clínico de IST.

## Conclusão

O conteúdo, a usabilidade e o desempenho funcional do aplicativo *IST Nurse* ^*®*^ apresentaram evidências de validade aceitáveis, atendendo à proposta de subsidiar o manejo clínico de IST por enfermeiros.

A técnica de grupo focal foi relevante no processo de definição de requisitos, sendo o Protocolo Clínico e Diretrizes Terapêuticas para Atenção Integral às Pessoas com Infecções Sexualmente Transmissíveis referencial teórico significativo para a construção do conteúdo do aplicativo, pois permitiu a elaboração de fluxos de informação com base em evidências científicas atualizadas.

As sugestões apresentadas pelos especialistas permitiram o aprimoramento do conteúdo e a redução de eventuais lacunas que possam surgir durante o uso do aplicativo, considerando as possibilidades e as demandas da rotina assistencial.

A tecnologia elaborada apresenta boas evidências de validade, tratando-se de proposta a ser somada à sistematização da assistência que facilitará a adoção de condutas mais coerentes no tocante ao acolhimento, à identificação e ao seguimento adequados no manejo clínico de IST.

# Translation: *IST Nurse* ^*®*^: desarrollo y evidencias de validez de una aplicación móvil como apoyo al manejo clínico de Infecciones de Transmisión Sexual*

**DOI:** 10.1590/1518-8345.7207.4316

**Authors:** Leilane Barbosa de Sousa, Ismael Moreira de Sousa, Edmara Chaves Costa, Ana Paula Fragoso de Freitas, Lydia Vieira Freitas dos Santos, Adriana Gomes Nogueira Ferreira

**Affiliations:** 1 Universidade da Integração Internacional da Lusofonia Afro-Brasileira, Redenção, CE, Brasil.; 3 Faculdade Rodolfo Teófilo, Curso de Graduação em Enfermagem, Fortaleza, CE, Brasil.; 4 Universidade Federal do Maranhão, Imperatriz, MA, Brasil.

**Keywords:** Aplicaciones Móviles, Tecnología de la Información, Manejo de la Enfermedad, Enfermedades de Transmisión Sexual, Atención de Enfermería, Salud Pública

## Abstract

**Objetivo:**

desarrollar y evaluar evidencias de validez para una aplicación móvil para apoyar al enfermero en el manejo clínico de las infecciones de transmisión sexual.

**Método:**

estudio metodológico realizado en cuatro etapas: análisis y definición de requisitos; definición de contenido; representación computacional con diseño del sistema; y codificación con pruebas y refinamiento. En las primeras etapas participaron enfermeros con experiencia en el tema y en la última, profesionales con formación en tecnologías de la información y las comunicaciones. El análisis de los datos se realizó mediante el cálculo del Índice de Validez de Contenido (IVC), considerando el valor mínimo de acuerdo de 0,78. Para confirmar la viabilidad del IVC se utilizó la prueba binomial mediante el *software* R. Las variables con p > 0,05 indicaron acuerdo entre los expertos.

**Resultados:**

el IVC fue de 0,98 para contenido, 1,0 para usabilidad y 0,85 para desempeño funcional, mostrando que la aplicación desarrollada tiene alta validez.

**Conclusión:**

se cree que la aplicación *IST Nurse* ^*®*^ representa una importante herramienta tecnológica para fortalecer el cuidado de enfermería basado en evidencias. Por lo tanto, se sugieren estudios de intervención.

## Destacados

**(1)** Falta de aplicaciones relacionadas con las ITS capaces de apoyar el manejo clínico.

**(2)** Fortalecimiento de la APS en la identificación y tratamiento temprano de las ITS.

**(3)** Producto útil para práctica clínica, formación y estandarización del actuar de los enfermeros.

**(4)** Reducción de errores en la conducta profesional, aumentando la calidad de la atención.

**(5)** Las tecnologías móviles democratizan el acceso a la información, con buen costo-efectividad.

## Introducción

El manejo clínico de las infecciones de transmisión sexual (ITS) sigue siendo un desafío para los profesionales de la salud, incluidos los enfermeros. La evaluación clínica ineficaz compromete la ruptura de la cadena de transmisión de estas infecciones, aumentando el número de casos. Mientras tanto, el razonamiento clínico es un elemento esencial en el proceso de toma de decisiones y favorece el diagnóstico y el tratamiento precoz, siendo estos factores esenciales para el control y la vigilancia de las ITS^1^ ^)-(^ ^2^.

La detección y el tratamiento temprano de las ITS reducen el riesgo de propagación, lo que resalta la necesidad de encontrar métodos para mejorar la atención a las personas con ITS, especialmente en el contexto del VIH/SIDA^3^. Por lo tanto, el enfermero puede beneficiarse de herramientas tecnológicas que puedan apoyar la atención de enfermería en ginecología, urología y andrología, cubriendo las diferentes poblaciones susceptibles a las ITS.

El uso de tecnologías de *eHealth* y su componente *mHealth* facilita el acceso y la difusión de información, permitiendo la interacción y el intercambio de conocimientos entre personas en diferentes ubicaciones geográficas^4^. La capacidad de generar, almacenar y compartir información pertinente a un ritmo rápido hace que los dispositivos tecnológicos sean herramientas atractivas y cada vez más utilizadas en la rutina de cualquiera que utilice estos recursos^5^.

Sin embargo, existe una brecha en la producción y disponibilidad de aplicaciones que aborden el manejo clínico de las ITS. En una revisión narrativa de la literatura realizada en enero de 2024 para aplicaciones móviles que abordan la temática ITS, se encontraron 38 aplicaciones para *Android* y tres para *iPhone Operating System* (iOS); no obstante, las tecnologías identificadas no están dirigidas al manejo clínico de las ITS.

Se cree que una aplicación móvil desarrollada con base en protocolos de atención integral y en algoritmos que orienten al enfermero hacia el razonamiento clínico podrá presentar evidencias de validez para apoyar la asistencia a personas con signos y/o síntomas de ITS. Así, el presente estudio tuvo como objetivo desarrollar y evaluar las evidencias de validez de una aplicación móvil para apoyar al enfermero en el manejo clínico de las ITS.

## Método

### Tipo de estudio

Se trata de un estudio metodológico basado en el desarrollo y análisis de evidencias de validez de una aplicación para dispositivos móviles sobre el manejo clínico de las ITS por parte de enfermeros. En resumen, este tipo de estudio permite investigar los métodos de obtención, sistematización y análisis de datos para la construcción de un instrumento^6^.

El estudio se desarrolló en dos fases. La Fase 1 fue el desarrollo de la aplicación en cuatro etapas: (1) análisis y definición de requisitos, (2) definición de contenido, (3) representación computacional con proyección de prototipo, y (4) codificación con pruebas y refinamiento. En la fase 2 se produjo la búsqueda de evidencias de validez de la aplicación, en términos de contenido, desempeño funcional y usabilidad.

### Lugar y período del estudio

El estudio se realizó en el municipio de Redenção, Ceará (CE), Brasil, entre septiembre de 2021 y junio de 2022.

### Población

El estudio incluyó a enfermeros que trabajan en la Estrategia de Salud de la Familia (ESF) (definición de requisitos para la aplicación y análisis de evidencias de usabilidad), enfermeros que trabajan en la docencia en el nivel de educación superior (análisis de evidencias de validez de contenido) y profesionales con formación en tecnologías de la información y las comunicaciones (análisis de evidencias de validez de desempeño funcional).

### Definición de la muestra

La invitación a participar del estudio se produjo inicialmente por medio de correo electrónico, ubicado a través de investigaciones en la Plataforma Lattes del portal del *Conselho Nacional de Desenvolvimento Científico e Tecnológico* (CNPq). El cuerpo del correo electrónico ofrecía un resumen que contenía el título de la investigación, objetivos y aspectos del método, así como el Formulario de Consentimiento Informado para otorgar el consentimiento. Esta estrategia, sin embargo, no tuvo éxito.

Por lo tanto, todos los participantes fueron seleccionados mediante muestreo no probabilístico, utilizando la técnica de red de referencia^7^. Esta técnica se dio a través de la selección de informantes clave llamados semillas, quienes ubicaron a algunas personas con el perfil necesario para la investigación. A estas personas nominadas se les pidió que nominaran nuevas personas con el perfil deseado. La comunicación pasó a ser vía *WhatsApp*, por donde se envió un video con la misma información contenida en el correo electrónico.

#### Fase 1: Desarrollo de la aplicación

Para definir a los enfermeros, se consideró el método de Diseño Centrado en el Usuario^8^, en el que se establece la participación/colaboración mutua entre los usuarios de la aplicación, considerándose eficaz en esta etapa el uso de la técnica de grupo focal. El número de participantes se definió entre seis y doce por grupo, según la referencia^9^.

A través de *WhatsApp* se contactó a los potenciales participantes y se les brindó orientación mediante el envío de audios sobre la propuesta del estudio, incluyendo cuestiones éticas para participar en la investigación.

Luego de 23 días de iniciar contacto con los potenciales participantes de la investigación, indicados por los informantes clave, fue posible seleccionar a 15 participantes. Dada la incompatibilidad de horarios disponibles para participar de una misma sesión grupal, fue necesario dividir a los profesionales en dos grupos, uno formado por nueve enfermeros y otro por seis.

#### Fase 2: Evidencias de validez de la aplicación

Para seleccionar los jueces para analizar evidencias de desempeño funcional, se consideraron algunas características definitorias a partir de la propuesta^10^, que fueron adaptadas para este estudio. Y para el análisis de las evidencias de usabilidad se retomó el contacto con los participantes de los dos grupos focales.

Respecto al número ideal de expertos, no existe consenso en la literatura. Así, fue definido con base en recomendaciones^7^, que proponen un número mínimo de seis expertos. Además de esta recomendación, se sugiere utilizar un número impar de expertos para evitar empates en las opiniones^7^. Esta recomendación fue adoptada para el análisis de evidencias de contenido y de usabilidad. Debido a la dificultad para encontrar expertos disponibles para analizar evidencias de desempeño funcional, y con base en una referencia específica para la evaluación de *software*, se encontró que este análisis ocurre bien con una cantidad de tres a cinco expertos^11^.

Se seleccionaron siete expertos para la evidencia de validez de contenido, tres para desempeño funcional y siete para el análisis de evidencias de usabilidad.

### Criterios de selección

#### Fase 1: Desarrollo de la aplicación

Se incluyeron enfermeros que trabajan en la ESF con al menos un año de experiencia, ya que, a partir de su trabajo diario con la persona/familia/grupo, tienen experiencia en la asistencia a personas con ITS. La determinación temporal está relacionada con la percepción de los autores sobre un período mínimo para que la experiencia en la práctica del manejo clínico permita propiedad suficiente para señalar las potencialidades y las debilidades de su actuación en este campo. Se excluyeron aquellos que se encontraban de vacaciones o ausentes por algún tipo de licencia.

#### Fase 2: Evidencias de validez de la aplicación

Fueron considerados expertos para el análisis de evidencias de contenido enfermeros investigadores/docentes con experiencia en el área de interés: salud sexual, ITS y/o tecnologías en salud. Fueron excluidos aquellos que no alcanzaron al menos cinco puntos en la aplicación de los criterios adaptados^10^, a saber: doctor o magíster con experiencia en el área de interés.

Para el análisis de evidencias de validez de usabilidad, participaron como expertos enfermeros que colaboraron en la etapa de definición de requisitos, en la fase 1 del estudio. Los criterios de inclusión y exclusión ya han sido explicados y justificados previamente.

Para el análisis de evidencias de desempeño funcional se incluyeron profesionales con formación y experiencia en las áreas de ingeniería de *softwares*, ciencias de la computación y análisis y desarrollo de sistemas, y se excluyeron aquellos que no cumplieron con los criterios adaptados^10^, a saber: tener título de Licenciatura en Tecnologías de la Información y las Comunicaciones; tener experiencia profesional en tecnologías de la información/comunicación/computación por un período mínimo de dos años; y tener experiencia en el desarrollo e implementación de sistemas.

### Fases del estudio

#### Fase 1: Desarrollo de la aplicación

Se siguieron cuatro etapas utilizando un método incremental e interactivo: (1) análisis y definición de requisitos; (2) definición de contenido; (3) representación computacional con diseño de sistema; y (4) codificación con pruebas y refinamiento^11^ ^)-(^ ^14^.

En el análisis y definición de requisitos se buscó comprender principalmente el conocimiento y las acciones de los enfermeros con relación al manejo clínico de las ITS. Esta etapa permitió acceder a las demandas de los enfermeros sobre lo que la aplicación debería ofrecer.

Después de identificar las demandas de los enfermeros, se buscó alianza con un equipo con experiencia en análisis y desarrollo de *software*, liderado por un profesor investigador doctor en Informática, de la *Universidade Federal do Maranhão*. El equipo y uno de los autores de la propuesta mantuvieron seis reuniones para definir las funcionalidades deseadas para la aplicación y recopilar requisitos para posteriormente utilizar la técnica de prototipado^15^. El mismo autor fue calificado por el equipo de socios para desarrollar la primera versión del prototipo de la aplicación utilizando la plataforma Figma^16^, en la que se definieron aspectos básicos y funcionales del sistema, como flujos de interacción, ubicación del botón, texto y mapeo de imágenes.

La etapa de definición de contenidos tuvo como objetivo recolectar la mejor evidencia científica sobre el tema propuesto, y se decidió utilizar como referencia base dos protocolos del *Ministério da Saúde* ^1^ ^),(^ ^17^. Estas son las referencias brasileñas más actuales sobre el tema.

La etapa de representación computacional con diseño del sistema consistió en formatear el contenido en un formato adecuado para su posterior codificación en Java. Esto dio como resultado la versión 1 del prototipo, que incorporó el cuerpo textual formulado a partir del análisis y definición de requisitos, siguiendo flujos lógicos de operación. El logotipo de la aplicación se creó con el *software* Canva^18^.

Después de evaluar la versión 1 por parte del equipo de desarrollo, la versión 2 se estructuró en base a proyectos puestos a disposición por la comunidad Figma^16^, siguiendo los estándares de diseño y de Interacción Humano-Computadora (IHC).

En la etapa de codificación con pruebas y refinamiento, la aplicación se desarrolló en módulos, permitiendo realizar pruebas de calidad a medida que se implementaba cada módulo. Esto facilitó la detección temprana de fallas para correcciones rápidas.

Para la codificación se utilizó el lenguaje de programación *Javascript* con el *framework React Native*, permitiendo la creación de aplicaciones para *Android* e *iOS*. El control de versiones se realizó con las herramientas *Git* y *Github*, y la instalación de paquetes se realizó con npm y Yarn.

Después de la codificación, la aplicación se instaló en un servidor *Expo* en la nube y se generó un *link* y un *Quick Response Code* para acceso. Esto permitió a los investigadores y expertos involucrados en el estudio acceder a la aplicación para probarla y perfeccionarla.

#### Fase 2: Evidencias de validez de la aplicación

Ocurrió en tres campos: contenido, desempeño funcional y usabilidad. Para definir los expertos de contenido, se adaptó un conjunto de requisitos propuestos^10^.

### Instrumentos utilizados y recolección de datos

#### Fase 1: Desarrollo de la aplicación

Las sesiones de grupo focal se desarrollaron de forma remota y sincrónica, y fueron grabadas vía correo electrónico institucional de uno de los autores para posterior recuperación de las declaraciones. Durante las reuniones se utilizaron preguntas desencadenantes por parte de un autor que moderó el momento, lo que hizo posible la discusión grupal.

El material grabado fue revisado para consideración, lo que permitió definir las necesidades de los profesionales y definir el contenido que debería componer la aplicación.

Identificadas las demandas de los enfermeros en el manejo clínico de las ITS, se buscó el contenido relevante en el manual y en el protocolo puestos a disposición por el *Ministério da Saúde* sobre el tema^1^ ^),(^ ^17^.

#### Fase 2: Evidencias de validez de la aplicación

Para la evidencia de validez de contenido se utilizó un instrumento adaptado^19^, que incluye ítems distribuidos en los siguientes bloques: objetivos y contenido (propósitos y metas o fines que se desean alcanzar por medio de la práctica con la tecnología) y estructura y funcionalidad (forma de presentar los lineamientos y practicidad en las funciones).

En el proceso de análisis de evidencias de validez de desempeño funcional se utilizó un instrumento basado en el Modelo de Calidad del Producto de la norma 25010 de la *International Organization for Standardization*/*International Electrotechnical Commission* (ISO/IEC), que especifica ocho características de calidad^20^.

Para analizar las evidencias de validez de usabilidad se utilizó la escala *Smartphone Usability Questionnaire* (SURE), con el objetivo de permitir al usuario final evaluar la aplicación de forma subjetiva observada directamente durante la prueba^21^.

Para cada ítem de los instrumentos y criterio evaluado se asignó una puntuación de 1 - inadecuado, 2 - parcialmente adecuado, 3 - adecuado, 4 - totalmente adecuado y 5 - no aplicable.

Los expertos participantes de las tres modalidades de proceso de análisis de evidencias validez recibieron los instrumentos en formulario vía correo electrónico y vía mensaje de *WhatsApp*. Se habilitaron espacios en los instrumentos para que los expertos realizaran comentarios y sugerencias sobre cada ítem evaluado. Después de 14 días, las respuestas de todos estaban disponibles.

### Procesamiento y análisis de los datos

Se realizó una reunión remota síncrona con cada grupo focal. Se presentaron los detalles del estudio y se formularon preguntas desencadenantes. Los discursos registrados mediante grabación de voz y textos fueron transcritos y procesados. Los discursos generados en las reuniones con los grupos focales fueron analizados siguiendo el método del discurso del sujeto colectivo^22^. Como herramienta de apoyo en este análisis se utilizó el *software* IRaMuTeq.

En el proceso de análisis se utilizó el Índice de Validez de Contenido (IVC) para medir el porcentaje de acuerdo para cada ítem de manera individual. El IVC se calculó mediante la suma de los ítems 3 (adecuado) y 4 (inadecuado) dividido por el número total de respuestas, considerándose como valor mínimo de acuerdo 0,78^23^. En caso de menor acuerdo, el punto se modificaría según sugerencias de los expertos.

Para confirmar la viabilidad del IVC, también se utilizó la prueba binomial procesando el comando binom.test (n° de éxitos, n° total, p=05) en el software R: aquí se probó si la proporción de éxito observada en la muestra pertenecía a una población con un determinado valor de *p*, en la que valores de *p* mayores a 0,05 indicaban acuerdo entre los jueces, no siendo estadísticamente inferior al 85%^24^.

### Aspectos éticos

La investigación fue aprobada por el Comité de Ética e Investigación de la *Universidade da Integração Internacional da Lusofonia Afro-Brasileira* con el dictamen número 4.889.214

## Resultados

IRaMuTeq generó una nube de palabras y un árbol de similitudes y, a partir de su evaluación, se formaron categorías temáticas siguiendo el método de análisis del discurso del sujeto colectivo. Estas categorías fueron importantes para el proceso de definición del contenido.

Se verificó, por medio de los grupos focales, que las clases más relevantes para el contenido de la aplicación son: dificultades relacionadas con el conocimiento del manejo por parte del profesional, reconocimiento de síntomas y falta de adherencia al tratamiento por parte de las parejas sexuales, uso de protocolos del *Ministério da Saúde* como consulta para el manejo clínico, y la practicidad y la facilidad que ofrecen tecnologías como las aplicaciones móviles para orientar el tratamiento adecuado de las ITS.

Estas clases sirvieron de base para la esquematización del contenido teórico de la aplicación, siendo los principales referentes el *Protocolo Clínico e Diretrizes Terapêuticas para Atenção Integral às Pessoas com IST* ^1^ y el *Caderno de Atenção Básica 18: HIV/Aids, Hepatites Virais, Sífilis e outras IST* ^17^, ambos del *Ministério da Saúde*.

Siguiendo el estándar *React Native*, se crearon 121 pantallas, organizadas en subcategorías de carpetas según las ITS y secciones de la aplicación. Por ejemplo, dentro de la carpeta “Dudas” hay subcarpetas correspondientes a cada infección. Esta estructura se definió para facilitar la organización de los archivos que componen el sistema, además de permitir su posterior mantenimiento.

La aplicación no utiliza ningún *back-end* para el procesamiento de datos, es solamente una aplicación de exposición de información y navegaciones. De esta forma, todas las pantallas desarrolladas son estáticas, presentando información relacionada con las ITS y la posibilidad de navegación (retroceder o avanzar en las pantallas). Además de las pantallas referidas al manejo clínico de las ITS, se desarrollaron otras sobre los creadores del proyecto, desarrolladores y referencias.

Después de todo el proceso de desarrollo, en total se escribieron aproximadamente 10.000 líneas de código, utilizando 54 imágenes. Luego, el cliente con la aplicación Expo - para pruebas y perfeccionamiento - puede, a través del *link* o *Quick Response Code*, abrir el sistema de la nube en su celular. Esto se hizo debido a la necesidad de ejecutar el sistema en un celular con sistema operativo *Android* y *iOS*, y este sistema operativo no cuenta con soporte y paquetes de instalación.

Tal como fue diseñada durante la creación del prototipo, la aplicación sigue un flujo que fue codificado en esta etapa. Este flujo obedece a comandos definidos en función del contenido utilizado, con inteligencia computacional aplicada al proceso de manejo clínico de las ITS.

La versión codificada del sistema, creada por medio del lenguaje de programación *Javascript* utilizado por el *framework React Native*, presenta una aplicación interactiva, siguiendo el flujo descrito anteriormente, siendo fácil de usar y navegar, que contiene información textual e imágenes, con base científica, fondo de pantalla oscuro con textos en color blanco y comandos en color verde.

Las pantallas iniciales de la aplicación se presentan en la Figura 1, que muestra el camino desde la pantalla *Home* hasta el abordaje terapéutico basado en el manejo clínico.

**Figura 1:** Flujo de pasos de la aplicación desde la pantalla *home* hasta el tratamiento indicado para un flujo vaginal. Redenção, CE, Brasil, 2022

### Análisis de las evidencias de validez de contenido

Los siete expertos que eran enfermeros y tenían experiencia docente en el nivel superior, como todos, participaron del grupo de investigación. Las áreas de actividad fueron salud sexual y reproductiva (n=2), salud familiar (n=3) y salud colectiva (n=2), y el tiempo promedio de trabajo en las áreas fue de 8,43 años. Todos obtuvieron una puntuación superior a cinco, referida a la suma de las puntuaciones atribuidas por el conjunto de requisitos.

Cuatro (57,2%) expertos tenían título de maestría y tres (42,9%) tenían doctorado. Uno de los expertos dirigió una disertación, cuatro dirigieron una monografía o un trabajo de conclusión de curso. Todos tenían publicaciones sobre temas de interés. Tres expertos combinaban docencia e investigación, y tres han publicado investigaciones sobre los temas de ITS y Tecnología en Salud.

Según la Tabla 1, la valoración de los expertos arrojó un promedio de 4 (totalmente adecuado) para la mayoría de los ítems evaluados, con un promedio de 3,71 para el ítem que evalúa la organización de la información. Por tanto, los ítems presentaron IVC con valores estadísticamente significativos, con una proporción de acuerdo entre jueces superior al 85% (*p*>0,05).

**Tabla 1:** Distribución de las respuestas de los expertos sobre el contenido presentado por la aplicación IST* *Nurse* ^*®*^ . Redenção, CE, Brasil, 2022

N°	Preguntas	mín^†^	pm^‡^	máx^§^	desv est^||^	IVC^¶^	p**
1	¿La aplicación mantiene coherencia con las necesidades de los enfermeros en cuanto al razonamiento clínico para el manejo de las ITS*?	4	4	4	0	1	1
2	¿La aplicación promueve la sistematización para el manejo clínico de las ITS*?	4	4	4	0	1	1
3	¿La aplicación puede circular en la comunidad científica en el área de manejo clínico de ITS*?	4	4	4	0	1	1
4	¿El material de la aplicación es adecuado para apoyar al enfermero durante el manejo clínico de las ITS*?	4	4	4	0	1	1
5	¿Está la información organizada de forma clara y objetiva?	3	3.71	4	0.49	1	1
6	¿La información presentada es consistente con la evidencia científica?	4	4	4	0	1	1
7	¿Existe una secuencia lógica del contenido propuesto?	4	4	4	0	1	1
8	¿Es el material apropiado en términos del nivel sociocultural del público objetivo propuesto?	4	4	4	0	1	1
9	¿Está la información bien estructurada en términos de concordancia y ortografía?	2	3.29	4	0.95	0,76	0,71
10	¿El estilo de escritura coincide con el nivel de conocimiento del público objetivo propuesto?	3	3.86	4	0.38	1	1
11	¿Las ilustraciones son expresivas y suficientes?	3	3.43	4	0.53	1	1
12	¿El número de pasos es adecuado?	3	3.86	4	0.38	1	1
13	¿El tamaño de los ítems y temas es apropiado?	3	3.86	4	0.38	1	1
14	¿Los temas retratan los aspectos clave a los que se debe prestar atención?	3	3.86	4	0.38	1	1
15	¿La aplicación proporciona a los enfermeros requisitos suficientes dirigidos a personas con ITS* para el manejo clínico de las ITS^*^?	3	3.86	4	0.38	1	1
16	¿La aplicación es adecuada para que la utilice cualquier enfermero que trabaje atendiendo a personas con ITS*?	3	3.86	4	0.38	1	1

*ITS = Infección de transmisión sexual; ^†^mín = Puntaje mínimo; ^‡^pm = Promedio; ^§^máx = Puntaje máximo; ^||^desv est = Desviación estándar; ^¶^IVC = Índice de validez de contenido; ^**^p = Prueba binomial

El único ítem que presentó baja proporción fue el nueve, que se refiere a la concordancia y la ortografía del contenido, que recibió un promedio de 3,29. Las consideraciones destacadas por los expertos se cumplieron mediante revisión y corrección ortográficas.

Los errores en la aplicación se atribuyeron a errores tipográficos y a que la plataforma no reconocía ciertos términos, lo que resultaba en la generación automática de palabras similares. Los expertos elogiaron la síntesis y exhaustividad del contenido, la sistematización y la agilidad de la aplicación, pero sugirieron más ilustraciones para mejorar el manejo clínico. También destacaron la eficiencia de la aplicación para abordar aspectos clave del contenido, con sugerencias para incluir información sobre las hepatitis y la ventana inmunológica.

El ajuste de la aplicación se realizó en base a estos comentarios. Con una desviación estándar que indica homogeneidad en la muestra y un Índice de Validez de Contenido (IVC) de 0,98, la aplicación muestra evidencias de validez y se considera útil para enfermeros que tratan con ITS.

### Análisis de las evidencias de validez de usabilidad

En esta etapa participaron siete expertos, todos enfermeros formados entre 2015 y 2020, con una experiencia media de 3,29 años en la ESF. La mayoría (85,71%) eran mujeres, mientras que solamente uno era hombre (14,29%). Seis eran especialistas (85,71%) y uno era magíster (14,29%).

La evaluación de la aplicación siguió la escala SURE^21^ para analizar evidencias de validez de usabilidad. Las respuestas de los expertos indicaron que algunas preguntas se consideraron positivas en la práctica, pero no estaban de acuerdo con las afirmaciones propuestas por la escala, y no fueron utilizadas para calcular el IVC.

Según la Tabla 2, la evaluación promedio de las preguntas de la escala antes mencionada osciló entre 3,57 y 3,86, demostrando homogeneidad en la muestra en cuanto a la idoneidad de la aplicación para el manejo clínico de las ITS. El IVC fue de 1,0, lo que refleja una evaluación positiva unánime de la aplicación por parte de los enfermeros. Las declaraciones de los expertos corroboraron los datos, mostrando satisfacción con la apariencia, usabilidad y contenido teórico de la aplicación, destacando su practicidad y capacidad para facilitar la práctica de los enfermeros con personas con ITS. 

**Tabla 2:** Distribución de las respuestas de los expertos de usabilidad de la aplicación IST* *Nurse* ^*®*^ . Redenção, CE, Brasil, 2022

N°	Preguntas	mín^†^	pm^‡^	máx^§^	desv est^||^	IVC^¶^	p**
1	Me resultó fácil ingresar datos en esta aplicación. Por ejemplo, usando código QR, lista de opciones, etc.	3	3.57	4	0.53	1	1
2	Cuando cometo un error, es fácil corregirlo.	3	3.57	4	0.53	1	1
3	La ayuda y los consejos proporcionados por la aplicación me parecieron útiles.	3	3.71	4	0.49	1	1
4	Fue fácil encontrar la información que necesitaba.	3	3.71	4	0.49	1	1
5	Me sentí al mando al usar esta aplicación.	3	3.71	4	0.49	1	1
6	Pienso que el tiempo que tomó completar las tareas fue adecuado.	3	3.71	4	0.49	1	1
7	Fue fácil aprender a usar esta aplicación. (Para responder a esta pregunta, solamente debe considerar usar la aplicación IST* *Nurse* ^*®*^ , no el proceso de instalación).	3	3.71	4	0.49	1	1
8	La secuencia de acciones en la aplicación corresponde a la forma en que normalmente las realizo, por ejemplo, el orden de los botones, campos de datos, etc.	3	3.57	4	0.53	1	1
9	Es fácil hacer lo que quiero usando esta aplicación.	3	3.71	4	0.49	1	1
10	Fue fácil navegar por los menús y pantallas de la aplicación.	3	3.86	4	0.38	1	1
11	La aplicación satisface mis necesidades.	3	3.86	4	0.38	1	1
12	Recomendaría esta aplicación a otras personas.	3	3.86	4	0.38	1	1
13	Incluso si tuviera prisa podría realizar tareas en esta aplicación.	3	3.86	4	0.38	1	1
14	La aplicación me pareció consistente. Por ejemplo, todas las funciones se pueden realizar de forma similar.	3	3.71	4	0.49	1	1
15	Es fácil recordar cómo hacer las cosas en esta aplicación.	3	3.86	4	0.38	1	1
16	Usaría esta aplicación con frecuencia.	3	3.71	4	0.49	1	1
17	La organización de los menús y comandos de acción (como botones y *links*) es lógica, lo que permite encontrarlos fácilmente en la pantalla.	3	3.86	4	0.38	1	1
18	Pude completar las tareas con éxito usando esta aplicación.	3	3.86	4	0.38	1	1
19	Me gustó usar esta aplicación.	3	3.86	4	0.38	1	1
20	La aplicación ofrece toda la información necesaria para completar las tareas de forma clara y comprensible.	3	3.86	4	0.38	1	1
21	La aplicación me pareció muy complicada de usar.	1	1	1	0	0	0
22	Los símbolos e ítems son claros e intuitivos.	3	3.71	4	0.49	1	1
23	Los textos me parecieron fáciles de leer.	3	3.86	4	0.38	1	1
24	La aplicación me pareció innecesariamente compleja. Tuve que recordar, investigar o pensar mucho para completar las tareas.	1	1	1	0	0	0
25	La terminología utilizada en textos, etiquetas, títulos, etc., es fácil de entender.	3	3.71	4	0.49	1	1
26	Necesitaría el apoyo de una persona para utilizar esta aplicación.	1	1.29	2	0.49	0	0
27	Me sentí cómodo usando esta aplicación.	3	3.71	4	0.49	1	1
28	La aplicación se portó como esperaba.	3	3.71	4	0.49	1	1
29	Usar esta aplicación me resultó frustrante.	1	1	1	0	0	0
30	Las diversas funciones de la aplicación me parecieron bien integradas.	3	3.57	4	0.53	1	1
31	Me sentí muy seguro al usar esta aplicación.	3	3.86	4	0.38	1	1

*ITS = Infección de transmisión sexual; ^†^mín = Puntaje mínimo; ^‡^pm = Promedio; ^§^máx = Puntaje máximo; ^||^desv est = Desviación estándar; ^¶^IVC = Índice de validez de contenido; **p = Prueba binomial

### Análisis de las evidencias de validez de desempeño funcional

En esta etapa participaron tres expertos, con formación en Ciencias de la Computación (n=2) y Sistemas de Información (n=1), siendo este último mujer. El año de formación es entre 2015 y 2021, con un promedio de 3,67 años de experiencia en el área de formación.

La Tabla 3 presenta el resultado de la evaluación de los expertos que realizaron el análisis de las evidencias de validez de desempeño funcional. Los promedios de evaluación de las preguntas estuvieron entre 3 y 4, lo que indica que los expertos coincidieron en que las funcionalidades de la aplicación son adecuadas o totalmente adecuadas. La desviación estándar lo corroboró, oscilando entre 0 y 0,58. El IVC fue estadísticamente significativo y, además, el valor de *p* para todos los ítems muestra una proporción de acuerdo del 100% entre los expertos.

**Tabla 3:** Distribución de las respuestas de los expertos de desempeño funcional de la aplicación IST* *Nurse* ^*®*^ . Redenção, CE, Brasil, 2022

N°	Preguntas	mín^†^	pm^‡^	máx^§^	desv est^||^	IVC^¶^	p**
1	¿Las funciones disponibles son suficientes para realizar las tareas propuestas por la aplicación?	4	4	4	0	1	1
2	¿Es comprensible el lenguaje utilizado en la aplicación?	3	3.67	4	0.58	1	1
3	¿La aplicación es coherente con el público objetivo al que está destinada?	4	4	4	0	1	1
4	¿La cantidad de información colocada en cada pantalla es adecuada para el público objetivo?	3	3.67	4	0.58	1	1
5	¿Los colores se utilizan con equilibrio, es decir, bien distribuidos, evitando así la contaminación visual?	3	3.67	4	0.58	1	1
6	¿Utilizando la aplicación es posible obtener información para lograr su objetivo principal?	3	3.67	4	0.58	1	1
7	¿Invita o instiga al usuario a un razonamiento sistemático?	4	4	4	0	1	1
8	¿Es coherente la información contenida en la aplicación?	4	4	4	0	1	1
9	¿Es atractiva para el usuario?	4	4	4	0	1	1
10	¿Es fácil entender los conceptos utilizados?	4	4	4	0	1	1
11	¿Las funciones son fáciles de aprender a usar?	4	4	4	0	1	1
12	¿Las presentaciones de las funciones de la interfaz (iconos, menús...) son fáciles de entender?	3	3.67	4	0.58	1	1
13	¿Es fácil de operar y controlar la operación?	4	4	4	0	1	1
14	¿La aplicación es consistente con lo esperado en relación a lo científicamente propuesto?	4	4	4	0	1	1
15	En caso de errores, ¿la aplicación permite la recuperación de los datos ya proporcionados?	3	3	3	NA^††^	1	1
16	¿Impide el acceso no autorizado, accidental o deliberado a programas de datos?	3	3.5	4	0.71	1	1
17	¿Promueve la integridad del usuario?	4	4	4	0	1	1
18	¿Es fácil encontrar fallas cuando ocurren?	3	3	3	NA^††^	1	1
19	¿Es fácil adaptarse a otros entornos sin aplicar otras acciones o medios distintos a los previstos para ello en el *software* considerado?	3	3.67	4	0.58	1	1
20	¿La aplicación es fácil de instalar?	3	3.67	4	0.58	1	1
21	¿Es lo suficientemente buena como para ser reemplazada?	4	4	4	NA^††^	1	1
22	¿Emite algún tipo de *feedback* cuando el usuario sigue una línea de razonamiento negativa?	4	4	4	0	1	1
23	¿Ofrece un resumen del desempeño del usuario al final del uso?	4	4	4	0	1	1

*ITS = Infección de transmisión sexual; ^†^mín = Puntaje mínimo; ^‡^pm = Promedio; ^§^máx = Puntaje máximo; ^||^desv est = Desviación estándar; ^¶^IVC = Índice de validez de contenido; ^**^p = Prueba binomial; ^††^NA = No aplicable

Respecto de las preguntas 15 y 17, considerando que uno de los expertos señaló la necesidad del registro del paciente en la aplicación, esto es algo que por decisión de los investigadores se desestimó por motivos de protección de datos del paciente, ya que la aplicación estará en uso en el teléfono celular del enfermero, y no en un dispositivo exclusivo del servicio. La aplicación tiene como objetivo orientar el manejo clínico de las ITS con respecto a las características propedéuticas evidenciadas durante la consulta de enfermería, lo que brindará apoyo al pensamiento crítico y reflexivo sobre la evaluación clínica. Sin embargo, otros datos personales deberán ser considerados por el profesional para su registro en los propios documentos del servicio.

Sobre las preguntas 16 y 23, se llevaron las consideraciones al equipo que desarrolló el sistema para incrementar una forma de acceso restringido para el profesional que tenga la aplicación *IST Nurse* ^*®*^ en su *smartphone*, además de ofrecer un resumen de los pasos seguidos por el usuario y la conducta adoptada.

De las preguntas 15 a 18 y 21 a 23, algunos jueces marcaron la opción “NA” como no aplicable. Esto transmite la información de que al evaluador no le gustaría responder esa pregunta por considerarla inadecuada para el objetivo del proceso de evaluación. En vista de esto, en las preguntas en las que un especialista seleccionó esta opción, éste no fue considerado como respondiente para el cálculo del IVC.

El IVC presentó un valor de 0,85, lo que representa un excelente nivel de acuerdo entre los expertos, y refleja que la aplicación cuenta con comandos y funciones adecuados o totalmente adecuados para cumplir lo que se propone.

Al final de la presentación de estos datos se presenta la Figura 2, en la que se muestran las sugerencias de los expertos para mejorar la aplicación, además de indicar si las sugerencias fueron aceptadas o no por los autores y de qué manera.

**Figura 2:** Sugerencias de los jueces y decisión de los autores sobre cambios en la primera plantilla de la aplicación. Redenção, CE, Brasil, 2022

Sugerencia	Decisión
**Análisis de evidencias de contenido**	
Ilustrar mejor la pantalla de inicio.	Aceptada. El logotipo se modificó para relacionarse mejor con el propósito de la aplicación.
Insertar más imágenes para apoyar al enfermero a mejor identificar/sospechar durante el manejo.	Aceptada. Se insertaron nuevas imágenes para la clínica presentada de las infecciones.
Mejorar la calidad de las imágenes.	
Mejorar la información sobre la conducta del manejo de la sífilis, especialmente en lo que respecta a la cicatrización serológica.	Aceptada. Se agregó contenido para orientar al enfermero sobre la necesidad de solicitar pruebas y conductas para el seguimiento de los casos de sífilis.
Abordar la ventana inmunológica en el tema de pruebas rápidas.	Aceptada. Se ingresó información para solicitar una nueva recolección dentro de los 30 días en caso de sospecha o resultados negativos.
Mejorar las imágenes de las pruebas rápidas.	Aceptada. Se insertaron nuevas imágenes para auxiliar en la interpretación de los resultados de las pruebas rápidas.
Insertar el tratamiento para las verrugas anogenitales.	Aceptada. Se ingresó la indicación del tratamiento.
**Análisis de evidencias de desempe**ñ**o funcional**	
Facilitar el proceso de instalación insertando en una de las tiendas.	No aceptada. La aplicación solamente estará disponible en las tiendas después de su aplicación en un estudio de evaluación de eficacia.
**Análisis de evidencias de usabilidad**	
Insertar un *link* en la aplicación que conduzca a la notificación.	No aceptada. Esta posibilidad se había considerado durante la creación del prototipo, pero debido a la orientación del equipo de desarrollo del *software*, no se incluyó.

## Discusión

El desarrollo de una tecnología dirigida a la vida diaria de los enfermeros necesita considerar las demandas que éstos presentan. Durante el desarrollo de la aplicación *IST Nurse* ^*®*^ , la estrategia de grupo focal *online* demostró ser efectiva para identificar temas que deben componer el contenido de la herramienta tecnológica, ya que permitió el diálogo con profesionales que actúan en diferentes ciudades y la verificación de necesidades y prioridades en el ámbito del manejo clínico de las ITS. Un estudio similar, que también utilizó el grupo focal *on line* como estrategia de diagnóstico situacional, destacó que esta estrategia contribuyó significativamente a la construcción de conocimientos en el área de la salud^25^.

Las demandas enunciadas en el diálogo con los enfermeros constituyeron el punto de partida para la elaboración del contenido de la aplicación. En este camino, el *Protocolo Clínico e Diretrizes Terapêuticas para Atenção Integral às Pessoas com Infecções Sexualmente Transmissíveis* (PCDT-IST)^1^ se configuró como un referente relevante para la base del flujo de datos en la aplicación, ya que presenta información basada en evidencia científica sobre las características de las ITS, y criterios para diagnósticos y manejos, incluyendo no solamente el tratamiento farmacológico, sino también directrices orientadas a la prevención y el control, esenciales para los cuidados de enfermería. Utilizando el PCDT-IST fue posible desarrollar contenido orientado a conducir el pensamiento clínico y definir conductas a partir de las demandas del paciente.

La aplicación *CuidarTech* ^*®*^ EnfPorElas, también desarrollada por enfermeros, consiste en una experiencia exitosa en la atención de enfermería a mujeres en situación de violencia sexual^26^. Esta tecnología, al igual que *IST Nurse* ^*®*^ , ofrece una lista de diagnósticos e intervenciones que aporta representación a la práctica de la atención de enfermería. Así, se observa la importancia de la inteligencia artificial como herramienta que ayuda al pensamiento clínico y a la toma de decisiones del enfermero, contribuyendo para su autonomía y poder de resolución de problemas.

Es necesario fomentar el uso de la IA en la atención en salud y, al mismo tiempo, garantizar que se desarrollen herramientas seguras y de calidad que beneficien al usuario. Por lo que es necesario el desarrollo de aplicaciones móviles basadas en un referente con soporte técnico-científico y el análisis de sus evidencias de validez a partir del análisis de profesionales experimentados^27^.

Los servicios móviles pueden proporcionar información más eficaz, sirviendo para ofrecer mejor conocimiento y ayudar al enfermero a la hora de realizar una consulta. Además, las redes pueden promover la difusión de orientaciones seguras sobre las ITS, contribuyendo a la reducción de conductas de alto riesgo^28^.

A pesar de la disponibilidad de protocolos clínicos y guías terapéuticas orientadas al manejo clínico de las ITS, es claro que esta práctica aún representa un desafío para los profesionales de la salud, incluidos los enfermeros. Una revisión integradora realizada a partir del análisis de 4 artículos y 5 disertaciones encontró deficiencias en el manejo clínico de la sífilis, especialmente en lo que respecta al conocimiento sobre criterios de diagnóstico y tratamiento, actitud ante la convocatoria de parejas sexuales y prácticas relacionadas con el abordaje de las parejas sexuales, y prescripción/administración de penicilina en unidades básicas de salud^29^.

Por eso, pensando en las dificultades que presentan los profesionales de la salud, incluidos enfermeros, la aplicación *IST Nurse* ^*®*^ fue diseñada para integrar información sobre la longitudinalidad de la atención integral a personas con ITS o con sospecha de ITS, incluso en casos de resultados negativos de pruebas rápidas. En una de sus pantallas, por ejemplo, la aplicación presenta el mandala de prevención combinada, pautas para solicitar pruebas no treponémicas en casos de pruebas treponémicas que resulten negativas o, aun, cuál debe ser la conducta en los casos en los que se sospeche de ITS incluso cuando las pruebas rápidas no confirmaron el diagnóstico.

Incluso partiendo de contenidos basados en un manual de referencia recomendado por el *Ministério da Saúde* de Brasil, el análisis de evidencias de validez de contenido, de usabilidad y de desempeño funcional por parte de los jueces es necesario e imprescindible. La experiencia de los expertos que participaron del presente estudio contribuyó en gran medida para la identificación de inconsistencias entre informaciones y/o vacíos en relación a las necesidades del enfermero en el manejo clínico de las ITS.

El IVC de los análisis de contenido (0,98), usabilidad (1,0) y desempeño funcional (0,85) presentó porcentajes aceptables, demostrando evidencias de validez para el uso de la aplicación por parte de enfermeros en el manejo clínico de las ITS. Sin embargo, buscando brindar al público objetivo la versión más segura y completa de la aplicación, se analizaron todas las sugerencias, ya que indagaban sobre conductas importantes en el cuidado diario de personas con ITS.

Considerando la sugerencia de uno de los jueces respecto a la ventana inmunológica para VIH, al revisar el *Protocolo Clínico e Diretrizes Terapêuticas* (PCDT)^1^, se encontró que existe indicación para solicitar una nueva prueba después de 30 días cuando haya sospecha clínica y/o epidemiológica. En vista de la relevancia del tema, se agregó este contenido a la aplicación.

Uno de los jueces indagó sobre el tratamiento de la sífilis en mujeres embarazadas ser en un plazo de tres semanas. El manejo rutinario de esta ITS considera tratar con 2,4 millones de unidades internacionales (UI) de Bencilpenicilina benzatínica, dosis única, en casos de sífilis reciente, siendo que algunos expertos recomiendan una dosis adicional de 2,4 millones de UI de penicilina G benzatínica una semana después de la primera dosis. Y, en casos de sífilis tardía, tratar con Bencilpenicilina benzatínica 2,4 millones de UI, una vez por semana, durante tres semanas^1^.

Se sabe que la atención en salud a las ITS se concentra principalmente en la Atención Primaria de Salud (APS), y que los profesionales que actúan en este contexto enfrentan desafíos en el manejo clínico por falta de preparación y desconocimiento^30^ ^)-(^ ^31^. Las aplicaciones pueden insertarse en la práctica del cuidado a personas con ITS, ya sea en un contexto preventivo o para diagnóstico, recordando siempre la importancia de la notificación y del tratamiento^32^ ^)-(^ ^33^.

Pensando en la notificación de las ITS, la aplicación aconseja al profesional realizar la notificación mediante el formulario propio de la unidad de salud, disponible en formato impreso. Al estar la aplicación en versión móvil, para *smartphones*, no sería útil facilitar *links* para acceder a los formularios de notificación, ya que están disponibles en un formato no modificable.

Hubo algunas dificultades para obtener una muestra más grande de expertos, aunque se enviaron invitaciones a 35 expertos para el análisis de evidencias de validez de contenido, 30 expertos para participar en el análisis de evidencias de validez de desempeño funcional y 34 para el análisis de evidencias de validez de usabilidad. Muchos no respondieron y algunos alegaron que no tenían tiempo para colaborar. Cabe señalar que hubo una espera de 45 días antes de la decisión de proceder con la muestra presentada.

Como limitación, cabe destacar que la escala SURE presenta preguntas con un sentido de respuesta negativo, lo que puede sesgar o inducir la respuesta del participante.

Se espera que un ensayo clínico randomizado evalúe la efectividad del uso de esta aplicación para apoyar las consultas de enfermería sobre ITS.

Antes de comercializar la aplicación, se propone formar una asociación con consejos profesionales para un posible catastro del profesional en la plataforma del *software* a partir de su registro. Se cree que de esta forma la aplicación quedará restringida a profesionales, evitando así la automedicación por parte de los pacientes.

Se considera que el producto de este estudio también será prometedor para la formación de enfermeros, ya que podrá ser utilizado durante actividades de enseñanza y extensión.

Además, se destaca la contribución a la práctica de los enfermeros que actúan en el contexto de la ESF, donde la mayoría de estos profesionales tienen más contacto con el manejo clínico de las ITS.

## Conclusión

El contenido, la usabilidad y el desempeño funcional de la aplicación *IST Nurse* ^*®*^ presentaron evidencias de validez aceptables, atendiendo a la propuesta de apoyo al manejo clínico de las ITS por parte del enfermero.

La técnica del grupo focal resultó relevante en el proceso de definición de requisitos, siendo el *Protocolo Clínico e Diretrizes Terapêuticas para Atenção Integral às Pessoas com Infecções Sexualmente Transmissíveis* un referente teórico significativo para la construcción del contenido de la aplicación, ya que permitió crear flujos de información con base en evidencia científica actualizada.

Las sugerencias presentadas por los expertos permitieron mejorar el contenido y reducir los vacíos que puedan surgir durante el uso de la aplicación, considerando las posibilidades y demandas de la rutina de cuidado.

La tecnología desarrollada presenta buenas evidencias de validez, por ser una propuesta a agregar a la sistematización de la atención que facilitará la adopción de conductas más coherentes en cuanto a la recepción, identificación y seguimiento adecuados en el manejo clínico de las ITS.
